# Rational design and implementation of a cucurbit[8]uril-based indicator-displacement assay for application in blood serum†
†Electronic supplementary information (ESI) available: Synthetic details, photophysical analysis, and further explanations on physicochemical models. See DOI: 10.1039/c9sc00705a


**DOI:** 10.1039/c9sc00705a

**Published:** 2019-06-04

**Authors:** Stephan Sinn, Eduard Spuling, Stefan Bräse, Frank Biedermann

**Affiliations:** a Karlsruhe Institute of Technology (KIT) , Institute of Nanotechnology (INT) , Hermann-von-Helmholtz-Platz 1 , 76344 Eggenstein-Leopoldshafen , Germany . Email: frank.biedermann@kit.edu; b Karlsruhe Institute of Technology (KIT) , Institute of Organic Chemistry , Fritz-Haber-Weg 6 , 76131 Karlsruhe , Germany; c Karlsruhe Institute of Technology (KIT) , Institute of Toxicology and Genetics (ITG) , Hermann-von-Helmholtz-Platz 1 , 76344 Eggenstein-Leopoldshafen , Germany

## Abstract

In this study, we report the first supramolecular indicator-displacement assay (IDA) based on cucurbit[*n*]uril (CB*n*) hosts that is operational in blood serum. Rational design principles for host–guest chemosensing in competitively binding media were derived through detailed mathematical simulations. It was shown that currently known CB*n*-based chemosensing ensembles are not suited for use in highly competitive matrices such as blood serum. Conversely, the simulations indicated that a combination of cucurbit[8]uril (CB8) and an ultra-high affinity dye would be a promising IDA reporter pair for the detection of Alzheimer's drug memantine in blood serum. Therefore, a novel class of [2.2]paracyclophane-derived indicator dyes for the host CB8 was developed that possesses one of the highest host–guest affinities (*K*_a_ > 10^12^ M^–1^ in water) known in supramolecular host–guest chemistry, and which provides a large Stokes shift (up to 200 nm). The novel IDA was then tested for the detection of memantine in blood serum in a physiologically relevant sub- to low micromolar concentration range.

## Abstract 2

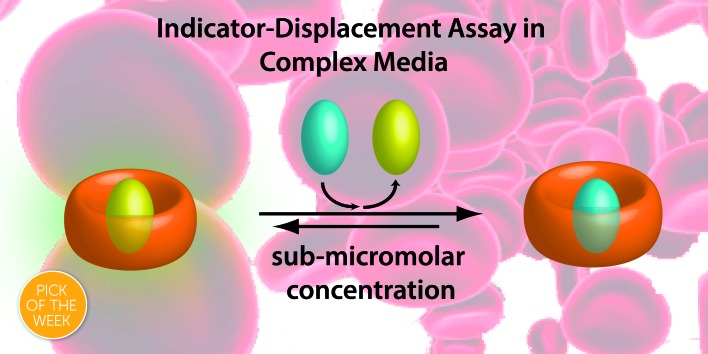
In this study, we report the first supramolecular indicator displacement assay (IDA) based on cucurbit[*n*]uril (CB*n*) host and a [2.2]paracyclophane derivative as indicator that is operational in blood serum.

## Background

**Fig. 1:**
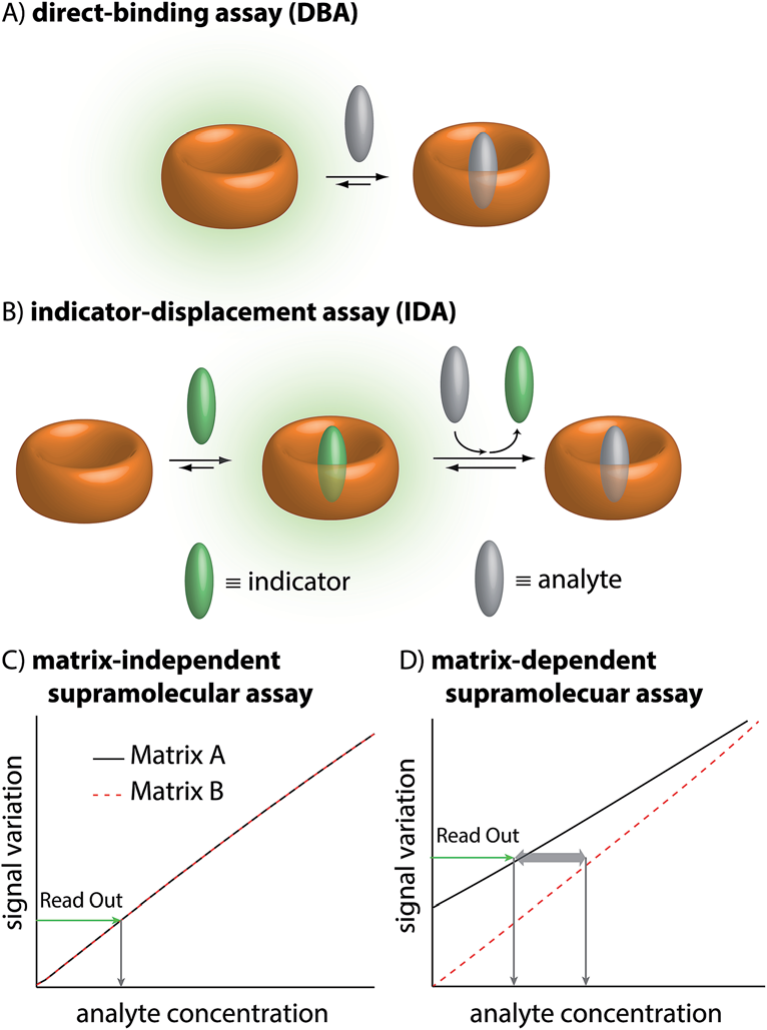
(A) Schematic representation of a direct-binding assay (DBA) utilizing a fluorescent host. (B) Schematic representation of an indicator-displacement assay (IDA) utilizing an indicator to precedingly form the reporter pair with a spectroscopically silent host and a subsequent displacement by the analyte. (C) Matrix effects perturb the application in complex media (blood) of unspecific binding assays by virtue of competitive binding. Left: Matrix-independent assay without perturbance by matrix variations (feasible with **MPCP**). (D) Matrix-dependent assay with known reporters.

It is one great promise of supramolecular chemistry that low-cost and chemically & thermally robust artificial receptors can complement contemporary antibody-based assays and chromatographic analytical methods in real-world sensing applications, *e.g.* for medical diagnostics.^1^–^4^ For instance, options for regular monitoring of drug levels in the blood of patients can assist the development of personalized medicine, taking individual drug bioavailability characteristics into account.^5^ Besides, quick and low-cost access to drug levels in biofluids can help medical staff to evaluate whether patients comply with the prescribed drug treatment. However, most synthetic supramolecular sensing methods face great challenges in biofluids, especially blood serum, because of the presence of salts, sugars, lipids, hormones, proteins, *etc.* that can act as competitive binders for the generally rather unselective artificial hosts (Fig. 1).^6^–^8^ In addition, strong absorbance and emission of blood over a wide wavelength range imposes additional difficulties.^8^ Lastly, for a real application, one would have to consider that the matrix could change (*e.g.*, the blood composition varies from one person to another). These changes could affect the sensor response independently of the concentration of analytes of interest.^9^

Thus, despite many proof-of-principle reports in organic solvents and in low-salt aqueous buffers,^1^,^10^–^14^ only a few supramolecular sensing systems are to date operational in blood serum in a practically relevant concentration range, *e.g.* the detection and quantification of alkaline metal cations (mM conc. range),^15^–^17^ of glucose (15 mM conc.),^18^ phosphate, pyrophosphate, AMP, or ATP (5 mM conc.)^19^ and of carnosine and homocarnosine (*ca.* 25 μM conc. in deproteinized human blood serum).^9^

**Fig. 2:**
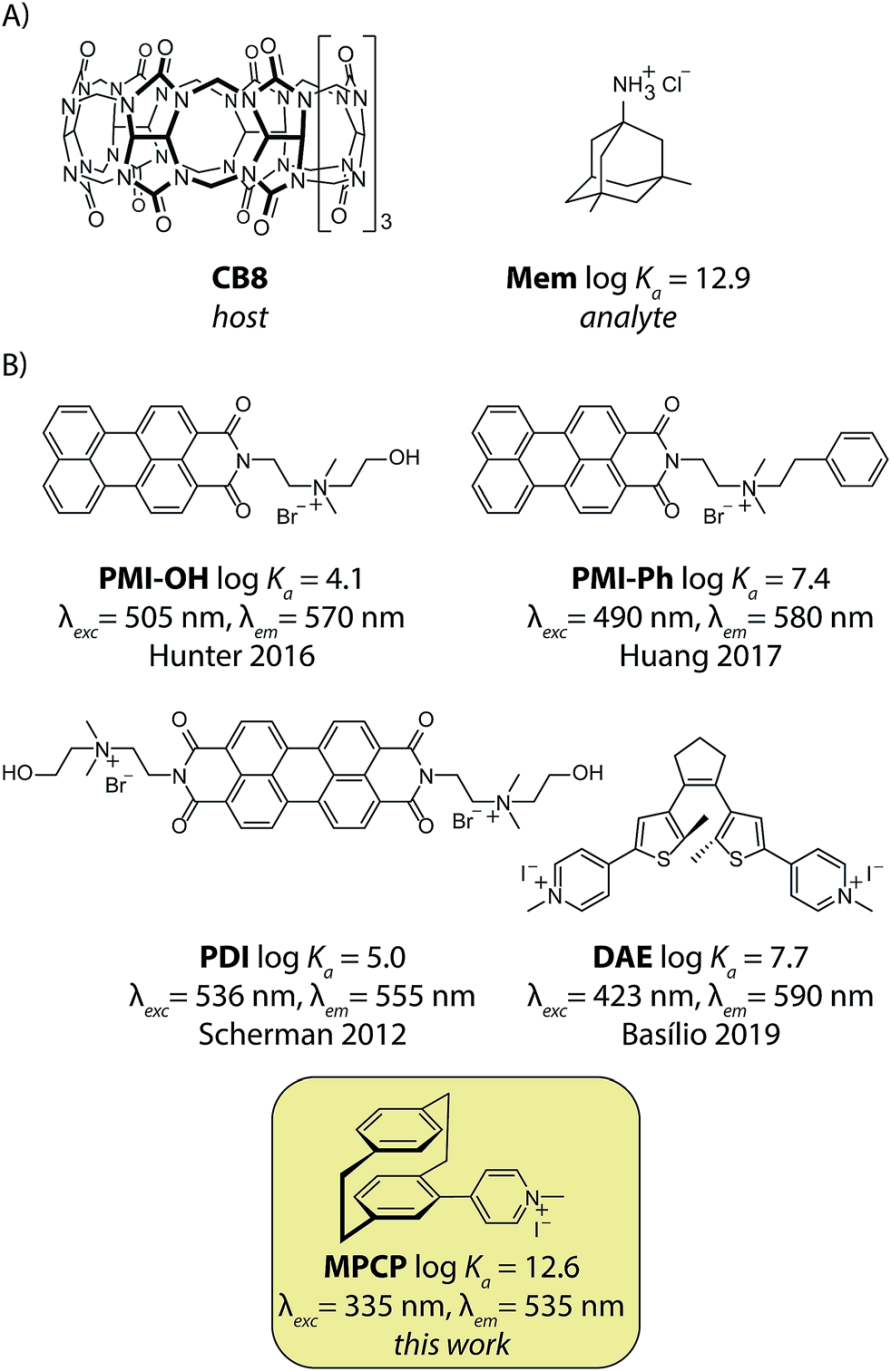
(A) The chemical structure of cucurbit[8]uril (**CB8**), the high-affinity unspecific host, and the Alzheimer's drug memantine hydrochloride (**Mem**), the analyte used in this study. (B) Published 1 : 1 binding fluorescent IDA dyes for CB8: **PMI-OH**,^39^**PMI-Phe**,^40^**PDI**,^41^–^43^ **DAE**^44^ and the novel high-affinity dye (**MPCP**) presented in this work. Their logarithmic binding constants with CB8 in aqueous media and the excitation and emission wavelength maxima of the CB8⊃dye complex are displayed.

The therapeutic window of many drugs is in the nanomolar to micromolar range. To illustrate, for Alzheimer's drug memantine (**Mem**)^20^ (Fig. 2A) the relevant plasma concentration range is between 0.03 and 1 μM.^21^,^22^ This implies that the binding affinity, *K*_a_, of an artificial receptor for the analyte to be detected in blood serum should reach at least 10^6^ M^–1^. Out of the many known supramolecular hosts, the macrocyclic cucurbit[*n*]urils (CB*n*, *n* = 5–10),^23^–^28^ see Fig. 2A, are particularly promising building blocks for the construction of chemosensors as they strongly bind a range of bio-relevant organic compounds.^3^,^29^–^35^ Three main sensing strategies that were adopted for CB*n*-based sensing studies are: (i) Direct-binding assays (DBA) where CB*n*-derivatives containing a remotely attached or framework-incorporated dye component yield a spectroscopic response upon binding of an analyte to the cavity (Fig. 1A).^3^,^36^,^37^ (ii) Associative-binding assays (ABA) for the detection of aromatic analytes. Note that the ABA, for which the binding cavity is formed by the host and a non-covalently incorporated reporter dye, is conceptually akin to a DBA.^3^,^29^,^30^ (iii) Indicator-displacement assays, IDAs, where an environment-sensitive indicator (*e.g.* an emissive or chromophoric reporter dye) is pre-complexed with CB*n* or its derivatives. If a host-binding analyte (*e.g.* memantine) is present in the medium, then the indicator is displaced, generating a readily measurable and quantifiable spectroscopic change (Fig. 1B).^1^,^3^,^10^–^13^,^38^

Regardless of the sensing method chosen, one will generally aim to obtain a linear signal–concentration response curve with a large slope, and which is not affected by naturally occurring sample-to-sample differences in the matrix composition in virtue of competitive interactions (Fig. 1C). In contrast, there are unacceptable large errors when correlating the measured signal with an apparent analyte concentration if the chemosensor is affected by the matrix and by sample-to-sample matrix variations (Fig. 1D).

In this contribution, we will show that only the IDA format and not a DBA holds promise for sensing in blood serum at physiologically relevant concentrations using high-affinity but unselective cucurbit[*n*]uril-based chemosensors. Moreover, it will be discussed that ultra-high-affinity indicator dyes for CB8 are needed to enable sensing in blood serum. Based on these guidelines, the first member of a [2.2]paracyclophane-based dye family is introduced and its utility for memantine-sensing in blood serum is demonstrated.

## Results and discussion

### General considerations for a memantine-sensing assay in complex media containing competitive binders

Memantine is an interesting starting point for establishing a drug-monitoring assay in blood serum because patients with dementia often fail to comply with the prescribed medication regimens.^45^ A large variance of memantine concentrations was found in clinical studies, which justifies regular monitoring of memantine concentration in order to reduce adverse drug effects such as confusion.^46^ However, the chromatographic methods used for the quantification of memantine require time-consuming sample preparation procedures;^47^,^48^ such assays are not suitable for regular use in point of care units.

**Fig. 3:**
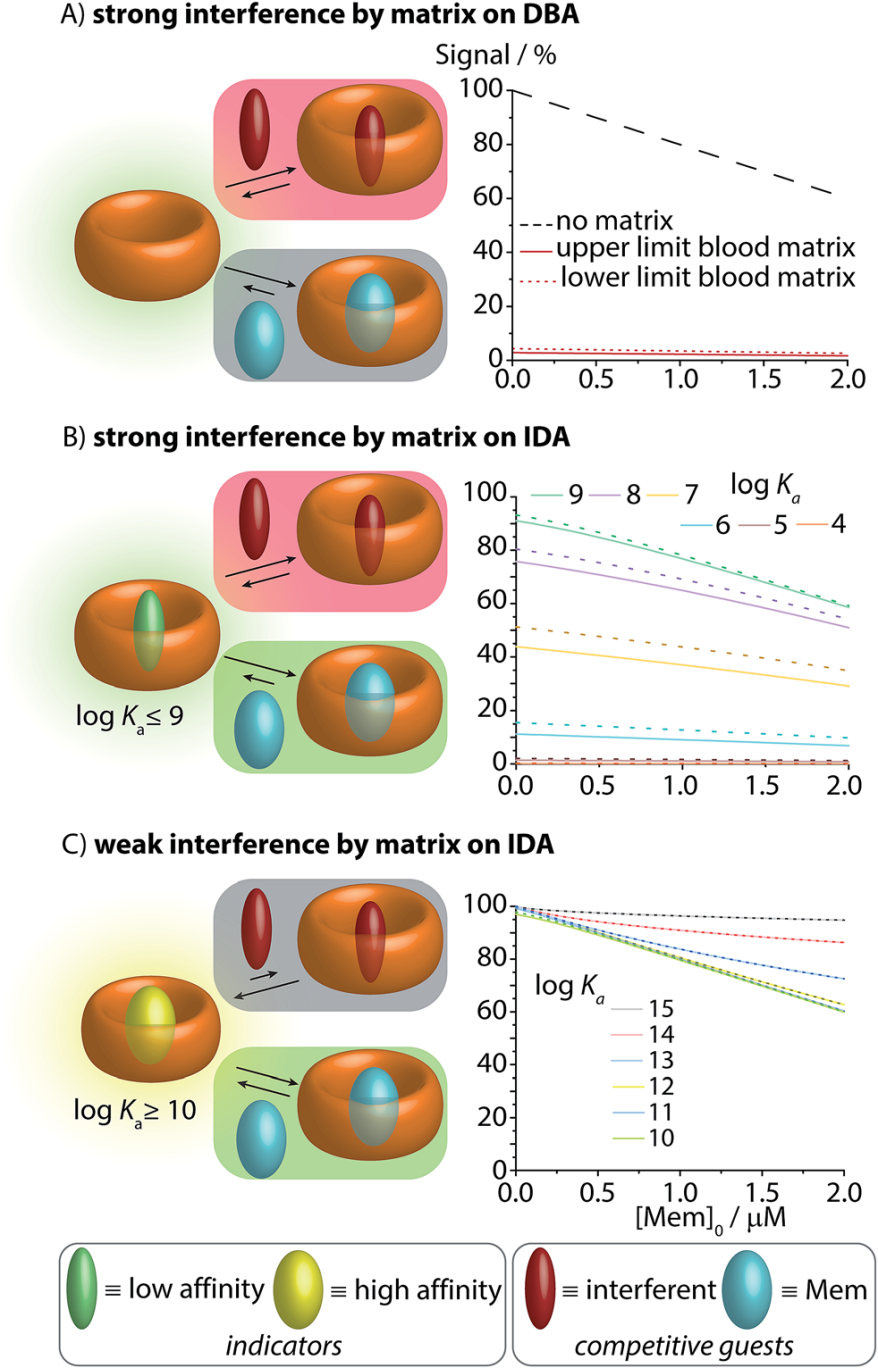
Simulations of the effect of blood matrix interferents on the CB8–memantine binding assay performance, comparing the lowest concentration limits of the interferents (dashed lines) and the highest limit (solid line) for blood serum matrices. See text and ESI† for details. (A) CB8-based direct-binding assay in the absence and presence of interferents in the matrix. (B) IDA featuring indicator dyes with log *K*_a_ ≤ 9 for CB8. (C) IDA featuring indicator dyes with log *K*_a_ ≥ 10 for CB8.

In the first step, we set to analyze and compare in detail the different sensing assay formats by taking use of mathematical simulations of binding isotherms. We wondered whether a direct-binding assay (Fig. 3A), utilizing a chromophoric CB8 derivative whose emission signal is quenched (or enhanced) upon analyte binding, would be the simplest route for the detection of spectroscopically silent analytes such as memantine. Indeed, the desired linear signal–concentration response curve is obtained in this assay type *if* rather “innocent” matrices such as water or low-salt buffers are considered as the medium (Fig. 3A, black dashed line). However, the low binding selectivity of CB*n* – and many other synthetic hosts – is the crucial limiting factor.^3^ For instance, endogenously present metabolites in blood such as amino acids Phe, Trp, Lys, Arg and ornithine (each occurring in the range of 20 to 200 μM)^49^ and hormones, *e.g.* cortisol (0.3 to 1.1 μM)^47^ are strong CB*n*-binders, see Fig. 6 for details.^3^,^33^,^35^ Besides, cations, such as Na^+^, K^+^ and Ca^2+^ which are present in the high millimolar range in urine^50^ and blood serum,^51^ show millimolar to micromolar affinities for CB*n*.^52^,^53^

When carrying out the mathematical binding isotherm simulation for DBA in the *simultaneous* presence of nine serum components that are binders for CB8, it becomes clear that a CB8-chemosensor would be nearly quantitatively complexed by the interferents (Fig. 3A, see ESI† for details). Therefore, the presence or absence of the target analyte memantine would only yield a very small spectroscopic response with a much smaller slope compared to the situation without a competitively binding matrix. Moreover, the response curves are also sample-dependent (solid and dotted red lines in Fig. 3A and in the zoom-in shown in Fig. S1 and S2 see ESI†), which is problematic because the absolute and relative concentration of blood components varies both from patient to patient and during time. The simulated results for the DBA in the presence of competitively binding matrix components are therefore akin to the undesirable matrix-dependent situation sketched in Fig. 1D. The direct-binding assay format appears to be not useful for application in blood serum when utilizing unselectively binding CB*n*-type hosts.

### Performance of an IDA in the presence of competitive binders

We then turned our attention to the IDA format with CB8–dye combinations as the chemosensing ensemble for the detection of memantine in blood serum.

Mathematical simulations were carried out to analyse the behaviour of CB8⊃dye chemosensing ensembles of varying binding affinity (log *K*_a_ = 4 to 15) in a competitively binding matrix upon increase of the memantine concentration (Fig. 3B and C). The following general observations were made: for a low affinity indicator dye (log *K*_a_ ≤ 9), both interferents and the analyte cause a significant amount of dye displacement (Fig. 3B). Specifically, it is clear that CB8-indicator dyes with log *K*_a_ ≤ 7 are generally not expected to be of use for IDA sensing applications in blood serum because the CB8⊃dye chemosensing ensemble is ≥50% decomplexed by the interferents. For log *K*_a_ ≤ 8 to 9, the chemosensing integrity is near 80% and 90%, respectively, which may be sufficiently high to conduct an IDA in blood serum. However, significant sample-to-sample matrix variation influences have to be expected in particular in the low but physiologically most relevant memantine concentration range, preventing an accurate concentration determination of memantine (Fig. 3B and zoom-in shown in Fig. S4–S6†). In short, the IDA in blood serum based on CB8⊃dye combinations with an affinity of log *K*_a_ ≤ 9 will likely result in undesirable signal–response curves similar to those shown in Fig. 1D.

Conversely, for an ultra-high affinity dye, both the interferents and the analyte will not appreciably displace the dye, and thus, there will be no undesirable matrix effects but also only a weak signal change in the presence of the analyte. Specifically, for log *K*_a_ ≥ 14, the degree of CB8⊃dye complexation is nearly quantitative but now the simulated signal response curves become too flat (Fig. 3C). Between the borders of too weakly and too strongly binding indicator dyes, one suspects to find an affinity range for which the matrix effects are small, but for which the signal–response curve in the presence of the analyte is linear and steep. Indeed, the “sweet spot” is reached for indicator dyes with a log *K*_a_ range of 10–13. Under these circumstances, a linear, steep signal–response curve is seen that is unaffected by matrix effects and sample-to-sample variations. This situation is akin to the ideal supramolecular sensing curve depicted in Fig. 1C; see also ESI† for numerical values.

Unfortunately, contemporarily known 1 : 1 binding emissive indicator dyes for CB8 possess maximal affinities in the range of 10^4^ < *K*_a_ < 10^8^ M^–1^ in water, see Fig. 2B, and therefore are inside the undesirable low-affinity range. We therefore figured that first a new emissive indicator dye for CB8 has to be designed that possesses an affinity within the predicted ideal range of 10^10^ to 10^13^ M^–1^ in water.

### Design of a novel picomolar indicator dye for CB8

**Fig. 4:**
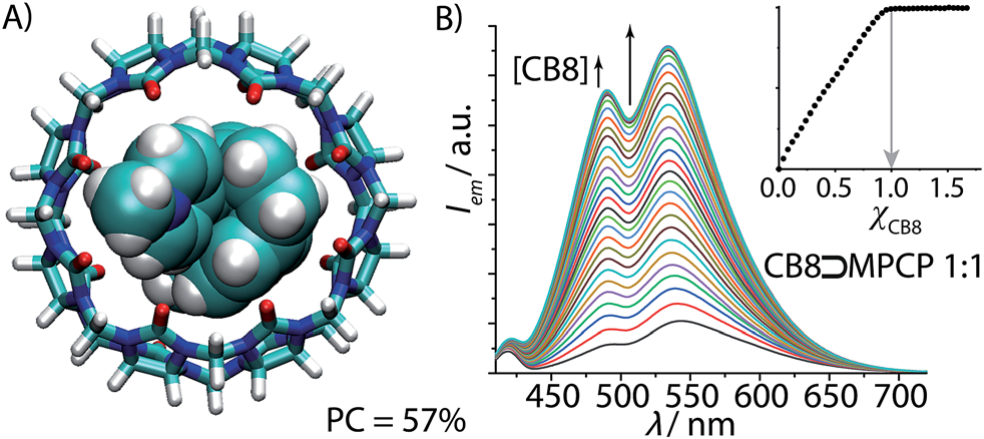
(A) Geometry-optimized DFT model of the ground-state of CB8⊃**MPCP** featuring an ideal packing coefficient (PC) of 57%. (B) Fluorescence spectra of **MPCP** (*λ*_exc_ = 335 nm, *λ*_em_ = 535 nm, *c* = 1.9 μM) in water with increasing CB8 concentration (0–3.1 μM). The obtained binding isotherm (inset) allows us to deduce the stoichiometry (1 : 1) and the assessment of a very strong binding (log *K*_a_ ≥ 9) (for accurate determination of *K*_a_ see Fig. 5 and Table 1).

Screening the tabulated thermodynamic binding parameters of a large number of CB8 guests showed that typical aromatic components (*e.g.* typical chromophores) and cyclic and acyclic aliphatic species (*e.g.* hydrocarbons, terpenes, steroids) do not exceed *K*_a_ values of ∼10^9^ M^–1^.^3^ In contrast, suitable ultra-high-affinity binding moieties for CB8 are spherical in shape such as metallocene complexes and adamantyl-species (*e.g.* memantine) and bind the host in a 1 : 1 stoichiometry. Unfortunately, they are themselves not emissive and it remains unclear how reporter dyes that have sufficient photophysical properties for sensing in blood serum (*e.g.* signal response factors, excitation wavelength and large Stokes shift) can be constructed from those moieties. We therefore searched for alternative spherically shaped compounds that would be inherently chromophoric and emissive. Inspired by the known, unique 2 : 1 homo- and 1 : 1 : 1 hetero-ternary complex formation of two aromatic moieties in CB8, we rationalized that two covalently tied aromatic rings, as found in [2.2]paracyclophane (PCP), should feature as a strong binding motif for CB8. In order to render the PCP moiety emissive, the installation of a pyridinium moiety onto the PCP backbone was carried out, targeting the known charge-transfer type emission mechanism of folded (phenylalkyl)pyridines.^49^ Besides, the positively charged pyridinium moiety would also ensure aqueous solubility and strengthen the binding affinity.^25^,^26^,^28^,^54^ We therefore prepared methyl-pyridinium-paracyclophane (**MPCP**, see Fig. 2B) as its iodide salt by a sequence of bromination of [2.2]paracyclophane, Pd-catalyzed cross-coupling with 4-pyridyl boronic acid and subsequent quaternization in an overall yield of 19%, see the ESI† for details.^55^ Indeed, molecular modelling (see Fig. 4A and ESI† for details) indicates a snug fit of the PCP moiety of **MPCP** in the CB8 cavity, arriving at a packing coefficient (PC) of 57%, which is spot-on in the ideal packing regime of (55 ± 9)% for supramolecular host–guest complexes.^56^,^57^ It is worth noting that **MPCP** is largely selective for the larger host CB8 over the smaller CB7 variant; the latter cannot engulf the PCP moiety but only the smaller pyridinium moiety (see ESI†).

### Experimental evaluation of CB8⊃**MPCP** host–guest interactions

In the next steps, the binding properties of **MPCP** with CB8 were systematically evaluated. First, the CB8⊃**MPCP** binding geometry was investigated by ^1^H NMR experiments in D_2_O. The ^1^H NMR spectrum of **MPCP** shows two sets of aromatic peaks at 8.15–8.75 ppm (2 × 2 protons of the pyridinium core) and at 6.53–6.85 ppm (7 PCP protons), see Fig. S18.† The aliphatic protons are at 4.38 ppm (CH_3_-*N*py moiety) and at 2.57–3.40 ppm (2× –CH_2_CH_2_– moieties of PCP). Upon addition of CB8, the aromatic and aliphatic PCP protons experienced a large upfield shift, indicating encapsulation of the PCP moiety inside the cavity of CB8. The pyridinium proton signals were only slightly upfield shifted, while the methyl-pyridinium proton signals remain rather unaffected. These findings, which are in agreement with molecular modelling results (Fig. 4A), confirm that the [2.2]paracyclophane moiety of **MPCP** is encapsulated inside the CB8 cavity, while the methyl-pyridinium group is located near the portal region of the host.

**Fig. 5:**
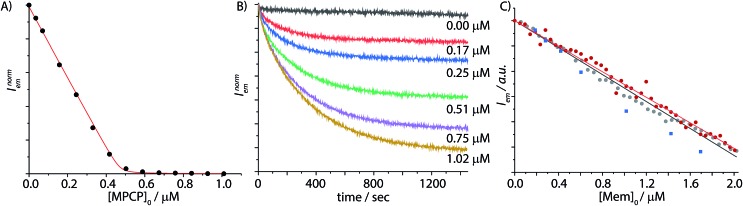
(A) Fluorescence-titration experiment derived binding isotherm of the displacement of berberine chloride (BC) from the CB8⊃BC_2_ (*λ*_exc_ = 462 nm, *λ*_em_ = 548 nm, *c*_CB8_ = 0.6 μM, *c*_BC_ = 59.2 μM) by **MPCP** (0–1 μM) in PBS. The data were fitted to a competitive binding assay, see ESI† for details. The logarithmic binding constants of CB8⊃**Mem** were determined to be log *K*_a_ = 12.59 (water) and log *K*_a_ = 10.88 (PBS) from ten repetitive titration experiments at *T* = 25 °C. (B) Fluorescence based kinetic traces at varied concentrations of **Mem** (average of triple replica is depicted) of the reversed indicator-displacement assay (RIDA) in PBS, in which the reporter pair CB8⊃**MPCP** (*λ*_exc_ = 335 nm, *λ*_em_ = 535 nm, *c* = 9.9 μM) is injected into a **Mem** spiked solution. Concentrations of **Mem** for each curve (top to bottom, black to yellow): 0, 0.17, 0.25, 0.51, 0.75, and 1.02 μM at *T* = 25 °C. (C) Fluorescence based IDA of CB8⊃**MPCP** (*λ*_exc_ = 335 nm, *λ*_em_ = 535 nm, *c* = 9.7 μM) with increasing concentration of **Mem** (0–2 μM) in PBS, (grey dots) with a linear calibration curve, *R*^2^ = 0.999 (black line) and in human serum (red dots) with a linear calibration curve, *R*^2^ = 1.000 (red line). Determination of unknown **Mem** concentrations in human serum (blue squares) based on the pre-determined calibration curve, *T* = 25 °C.

The aqueous solutions of **MPCP** are emissive in the visible area of the electromagnetic spectrum (*λ*_max_ ∼ 550 nm, see Fig. 4B) and possess a large Stokes shift (up to 200 nm) in polar and nonpolar solvents, see Table S1 and photophysical spectra in the ESI.† Investigation of the absorbance and emission properties of **MPCP** with respect to solvent polarity yielded a photophysical response that is indicative of a charge-transfer type emission, in agreement with the large Stokes shift in water (see ESI† for details). Upon addition of CB8, the fluorescence spectra display a 6.8 fold increase in the emission intensity with a small hypsochromic shift. The titration curve clearly shows a very strong **MPCP**–CB8 binding, supporting the expected 1 : 1 binding stoichiometry (Fig. 4B) that was verified by absorbance-based titration and ESI-MS experiments. Because the titration curves were very steep even at nanomolar concentrations of dye and host, *i.e.* (*K*_a_ ≫ 10^9^ M^–1^), a competitive binding method was adopted for determining the affinity of the CB8⊃**MPCP** complex (Fig. 5A). To this end, a measurement protocol and mathematical fitting routine for berberine chloride (BC) as an indicator and competitive 2 : 1 binder for CB8 was established that can be generally employed to determine the affinity of strong CB8 binders in aqueous media (*e.g.* for amantadine, memantine see the ESI†). For the CB8⊃**MPCP** complex, a remarkably high binding affinity of *K*_a_ = 3.89 (±0.99) × 10^12^ M^–1^ was obtained in deionized water (Table 1). Thus, **MPCP** is to date one of the strongest binding guests for CB8 in general and the strongest binding 1 : 1 complex forming indicator dye for CB8. Indeed, even in phosphate buffer saline (PBS) as a model for a biofluid consisting of 137 mM NaCl, 2.7 mM KCl, 10 mM Na_2_HPO_4_ and 2 mM KH_2_PO_4_, the binding affinity of **MPCP** for CB8 remained extremely high, 7.55 (±1.68) × 10^10^ M^–1^. **MPCP** is therefore one of the strongest binding guests known to date for CB8.^53^ Analogously, the binding affinities of **Mem** were determined to be *K*_a_ = 8.28 (±0.38) × 10^12^ M^–1^ in deionized water and 1.33 (±0.47) × 10^11^ M^–1^ in PBS using the competitive binding method. For comparison, Isaacs and co-workers found a value of *K*_a_ = 4.3 × 10^11^ M^–1^ in 50 mM NaO_2_CCD_3_ (pD = 4.75) as determined by competitive NMR experiments.^58^ We believe that the PCP dye class can be of further use for determining the binding strength of spectroscopically silent, ultra-high affinity guests for CB8 such as diamantane^53^,^59^ in a single fluorescence-based titration experiment, complementing current multi-step competition NMR-titration methods.

**Table 1:** Thermodynamic data for host–guest interactions of **Mem** and **MPCP** with CB8

Complex	Medium	*K*_a_ ^*a*^	log *K*_a_^*b*^
CB8⊃**MPCP**	Water^*c*^	3.89 (±0.99) × 10^12^ M^–1^	12.59 (±0.02)
	PBS^*d*^	7.55 (±1.68) × 10^10^ M^–1^	10.88 (±0.10)
CB8⊃**Mem**	Water^*c*^	8.28 (±0.38) × 10^12^ M^–1^	12.92 (±0.02)
	PBS^*d*^	1.33 (±0.47) × 10^11^ M^–1^	11.12 (±0.15)

^*a*^Binding affinity obtained at *T* = 25 °C.

^*b*^Decadic logarithm of *K*_a_.

^*c*^Experiments were performed in MilliQ water.

^*d*^Experiments were performed in phosphate buffered saline (PBS) consisting of 137 mM NaCl, 2.7 mM KCl, 10 mM Na_2_HPO_4_ and 2 mM KH_2_PO_4_.

### Development of a CB8⊃**MPCP**-based IDA for memantine detection in saline buffers and blood serum

The titration of CB8⊃**MPCP** with memantine was then carried out in phosphate buffer saline (PBS) as a first test whether an IDA can be set up for the detection and quantification of memantine in a “biological fluid”. It is worth noting that equilibration times can be very long (*e.g.* up to several days) for ultra-high-affinity receptor–ligand or host–guest systems, which can be a serious drawback for a sensing application.^60^–^62^ For the displacement of **MPCP** by **Mem** from CB8, we observed equilibration times of approximately 8 to 30 min upon increasing the **Mem** concentration from 170 nM to 1 μM (Fig. 5B). Fortunately, the equilibration times are well inside a practically convenient range for an analytical assay. The data (0.00 μM trace in Fig. 5B) also indicated that CB8⊃**MPCP** is sufficiently photostable (≥20 min continuous irradiation in the spectrofluorometer). We then experimentally measured the emission response upon increasing concentrations of **Mem**, covering a range from 0.05 to 2 μM, in PBS at a fixed CB8⊃**MPCP** chemosensor concentration of 9.7 μM. Pleasingly, the observed signal–concentration response curve was highly linear, *R*^2^ = 0.999, see Fig. 5C.

Encouraged by these results, we then turned our attention to blood serum as the practically most relevant but challenging biofluid for assay development. In addition to the competitive binding interferents present in blood serum, emission-based sensing applications in blood serum are further complicated by the strong absorbance and auto-emissive properties of this biological matrix. Fortunately, the novel indicator dye **MPCP** shows a much larger Stokes shift than previously reported IDA dyes for CB8, see Fig. 2B, assisting in reducing the emission background signal from the blood serum matrix. Besides, low cost disposable PMMA plastic cuvettes can be utilized with the CB8⊃**MPCP** reporter pair. Fortunately, the excitation maximum of CB8⊃**MPCP** around 335 nm coincides with a local absorbance minimum of unfiltered and filtered blood sera (Fig. S20†), enabling the development of the CB8⊃**MPCP** based IDA assay in this biofluid, see Fig. S27.† A highly linear calibration curve (*R*^2^ = 0.996) was obtained by the stepwise addition of 75 aliquots of memantine (final concentration of 0.12 μM to 8.3 μM) to bovine blood serum that was spiked with 9.9 μM CB8⊃**MPCP**, see Fig. S28.† It is worth noting that deviations from the linearity are to be expected for IDAs when the analyte concentration approaches that of the reporter pair. Indeed, Fig. S29† shows this behavior both for the simulated and experimentally observed calibration curve in human blood serum.

Also in human blood serum the analyte **Mem** could be quantified at a sub- to low micromolar concentration (Fig. 5C, calibration curve with *R*^2^ = 1.000), covering the physiological concentration range of memantine during Alzheimer's treatment. Moreover, we observed that a calibration curve obtained in PBS could be overlaid with the calibration curve obtained in human blood serum and in bovine blood serum, Fig. 5C & S30,† by shifting the *y*-axis offset to account for different absolute emission intensities originating from background emission and inner filter effects in the different media. Likewise, the influence of sample-to-sample matrix variation differences was tested by repeating the calibration curve measurement (0 to 2 μM memantine) in different bovine blood serum batches (Table S4†). The slopes of the memantine calibration curves were very similar (≤10% difference to the mean) between the three bovine blood serum batches tested, which agrees well with the predictions from the binding isotherm simulations (see Fig. 3C) and meets an important prerequisite for the development of a quantitative analytical memantine assay. Utilising the pre-determined calibration curves, we then succeeded in detecting unknown concentrations of memantine in spiked human blood serum with relatively high accuracy, see Fig. 5C. We believe that sample-to-sample differences caused by photophysical effects (*e.g.* inner filter effects) are the main cause for the remaining uncertainties when using the CB8⊃**MPCP** indicator pair. To emphasize, a memantine calibration curve obtained in pig blood serum showed a much steeper albeit still linear calibration curve (*R*^2^ = 0.999), Fig. S31.† It has to be noted that pristine bovine blood serum and pig blood serum not only differ in their molecular composition but also strongly in their cloudiness and optical properties (absorbance and emission spectra). Future efforts will be therefore directed towards the development of brighter **MPCP** derivatives that are excitable at ≥450 nm and that show higher emission on–off ratios upon CB8 binding in order to establish an emission-background independent memantine assay.

## Experimental

### General materials and methods

All solvents were used as received from Aldrich or Fluka without any further purification. The compounds were purified by column chromatography using silica gel 60 (230–400 mesh, Merck Millipore) as the stationary phase. ^1^H and ^13^C NMR spectra were recorded on a Bruker AM 400 or a Bruker Avance 500 spectrometer at room temperature. The ^1^H NMR chemical shifts (*δ*) are given in ppm and refer to residual protons on the corresponding deuterated solvent: chloroform (7.26 ppm in ^1^H; 77.0 ppm in ^13^C) and water (4.79 ppm in ^1^H; no signal in ^13^C). The assignments of the signal structure in ^1^H NMR were made by the multiplicity and for ^13^C NMR by DEPT90- and DEPT 135-spectra (DEPT = distortionless enhancement by polarization transfer) with + = primary or tertiary C-atom (positive DEPT-signal), – = secondary C-atom (negative signal) and C_q._ = quaternary C-atom (no signal). All deuterated solvents were used as received without any further purification. All coupling constants (*J*) are given in hertz (Hz). IR spectra were recorded on a FT-IR Bruker IFS 88 spectrometer. The compounds were measured as pure substances by the ATR technique (ATR = attenuated total reflection). The intensities of the bands were characterized as follows: vs = very strong (0–20% T), s = strong (21–40% T), m = medium (41–60% T), w = weak (61–80% T), and vw = very weak (81–100% T). Mass spectrometry (MS) experiments were performed on a Bruker Daltonics micro-Q-ToF II spectrometer equipped with an orthogonal electrospray (ESI) interface or on a Finnigan MAT 95 (EI-MS). The peaks are given as mass-to-charge-ratio (*m*/*z*). The molecule peak is given as [M]^+^ and characteristic fragment peaks are given as [M – fragment]^+^ or [fragment]^+^. The signal intensities are given relatively (in percent) to the intensity of the base signal (100%).

Absorption spectra were recorded on a Jasco V-730 double-beam UV-VIS spectrophotometer and baseline corrected. Steady-state emission spectra and time resolved emission profiles were recorded on a Jasco FP-8300 fluorescence spectrometer equipped with a 450 W xenon arc lamp, double-grating excitation, and emission monochromators. Emission and excitation spectra were corrected for source intensity (lamp and grating) and the emission spectral response (detector and grating) using standard correction curves. For spectral recording the automatic filter change of the FP-8300 was applied avoiding second order diffraction artefacts. Titration curves were recorded using a Jasco FP-8300 fluorescence spectrometer equipped with an ATS-827 automatic titration unit filled with the appropriate compound (host/guest) and subsequently corrected for dilution. All titration and kinetic experiments were carried out at *T* = 25 °C by using a water thermostatted cell holder STR-812, while the cuvettes were equipped with a stirrer allowing rapid mixing. For spectroscopy analysis in quartz cuvettes, suprasil (type 111-QS) emission cuvettes with a light path of 10 mm and dimensions of 10 × 10 mm from Hellma-Analytics were utilized. For titration experiments PMMA cuvettes with a light path of 10 mm and dimensions of 10 × 10 mm from Brand with a spectroscopic cut-off at 300 nm were utilized.

### DFT calculations

Density functional theory (DFT) calculations have been carried out in order to evaluate the ground state structure of the inclusion complex between CB8 and **MPCP** as well as CB7 and **MPCP**. DFT calculations were carried out utilizing the hybrid functional B3LYP with the standard valence basis set 6-31G(d,p) for C,H,N and O. The found stationary points possess no imaginary frequencies and therefore assumed to be true minima. All calculations were performed with the program package G16.B.01.^63^

### Synthesis of methyl-pyridinium-paracyclophane (**MPCP**)

PyPCP (50.0 mg, 180 μmol, see ESI† for synthesis) was dissolved in 1.5 mL dichloromethane in a one neck round bottom flask equipped with a magnetic stirrer. Subsequently, 1 mL of methyl iodide (2.28 g, 16.1 mmol) was added under ambient conditions resulting in a pale yellow solution. The crude mixture was allowed to react for 72 h, while two portions of methyliodide (1 mL) were additionally added after 24 h and 48 h. The crude reaction mixture was extracted three times with water. After the removal of water by lyophilization, **MPCP** was obtained as a yellow powder (42.1 mg, 98.5 μmol, yield: 54.7%). ^1^H NMR (500 MHz, D_2_O) *δ* = 8.76 (d, *J* = 6.4, 2H), 8.14 (d, *J* = 6.5, 2H), 6.86 (d, *J* = 11.5, 3H), 6.80–6.68 (m, 3H), 6.53 (d, *J* = 7.9, 1H), 4.38 (s, 3H), 3.45–3.35 (m, 1H), 3.25–3.06 (m, 5H), 3.03–2.94 (m, 1H), 2.62–2.52 (m, 1H). ^13^C NMR (126 MHz, D_2_O): *δ* = 144.6 (+, CH), 141.6 (C_q._), 140.6 (C_q._), 140.0 (C_q._), 138.8 (C_q._), 137.0 (+, CH), 135.9 (+, CH), 135.8 (C_q._), 133.7 (+, CH), 133.2 (+, CH), 132.4 (+, CH), 131.8 (+, CH), 129.5 (+, CH), 127.7 (+, CH), 47.2 (+, CH_3_), 34.6 (–, CH_2_), 34.3 (–, CH_2_), 34.2 (–, CH_2_), 33.4 (–, CH_2_) ppm. IR (ATR) *ν* = 3386 (vw), 3032 (vw), 2920 (vw), 2850 (vw), 2573 (vw), 2322 (vw), 2168 (vw), 2020 (vw), 1918 (vw), 1639 (vw), 1516 (vw), 1454 (vw), 1332 (vw), 1198 (vw), 1096 (vw), 949 (vw), 843 (w), 720 (vw), 642 (vw), 488 (vw) cm^–1^. HRMS (ESI, C_22_H_22_N^+^) calc. 300.1747, found 300.1737. EA (C_22_H_22_IN·5H_2_O) calc. C 51.06%, H 4.29%, N 2.71%; found C 50.90%, H 4.29%, N 2.81%.

### Photophysical studies

The electronic absorption spectrum of **MPCP** in water is displayed in Fig. S11.† The high energy transitions at 225 nm and 255 nm are ascribed to the π → π* transitions localized on the [2.2]paracyclophane scaffold. The low energetic transition peaking at 351 nm is assigned to an intramolecular charge-transfer π → π* transition (ITC) band from the [2.2]paracyclophane to the methylated pyridinium entity. **MPCP** displays room temperature emission peaking at 545 nm in aqueous solution. The acquired excitation spectrum is in good agreement with the corresponding absorption spectrum (Table 2).

**Table 2:** Summary of the photophysical parameters of **MPCP** and its inclusion complex with CB8 under ambient conditions in water

Technique	Quantity/unit	**MPCP**	CB8⊃**MPCP**
Absorbance	*λ*_abs,max_/nm	335	342
	*ε*_*λ*_max__/M^–1^ cm^–1^	7111 (±190)	5653 (±152)
	*λ*_isosbestic_/nm	366	
Fluorescence	*λ*_em,max_/nm	535	542

### Supramolecular host–guest binding studies

In order to obtain binding isotherms suitable for a meaningful fitting of the binding constant, competitive fluorescence titration experiments were carried out. Firstly, the binding constants of the complex formation of CB8⊃BC_2_ were determined by fluorescence titration experiments with a subsequent binding model on the basis of eqn (1)–(4) (*α* is the corresponding emission intensity factor fixed by the quantum yield ratio for *α*_HDD_ and typically results in 0 for *α*_D_) in very good agreement with literature values from Biczok *et al.* (see Table S3†).^64^ Secondly, the binding isotherms of ultra-high affinity guests such as **MPCP** and **Mem** were obtained by an IDA with CB8⊃BC_2_. The isotherms were fitted to a binding model derived from eqn (1) to (3) and (5) to (7) and the value of the corresponding *K*_a_ determined as the mean (±standard deviation) of ten independent titration experiments (see ESI† for titration data).

1 *I*emsignal = *α*_HD_[HD] + *α*_HDD_[HDD] + *α*_D_[D]

2
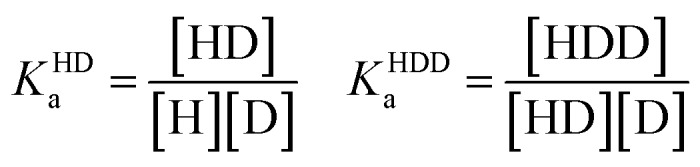

3 [D]_0_ = [D] + [HD] + 2[HDD]

4 [H]_0_ = [H] + [HD] + [HDD]

5
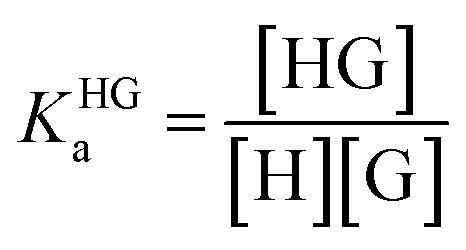

6 [G]_0_ = [G] + [HG]

7 [H]_0_ = [H] + [HD] + [HDD] + [HG]

### Mathematical simulations of binding isotherms

**Fig. 6:**
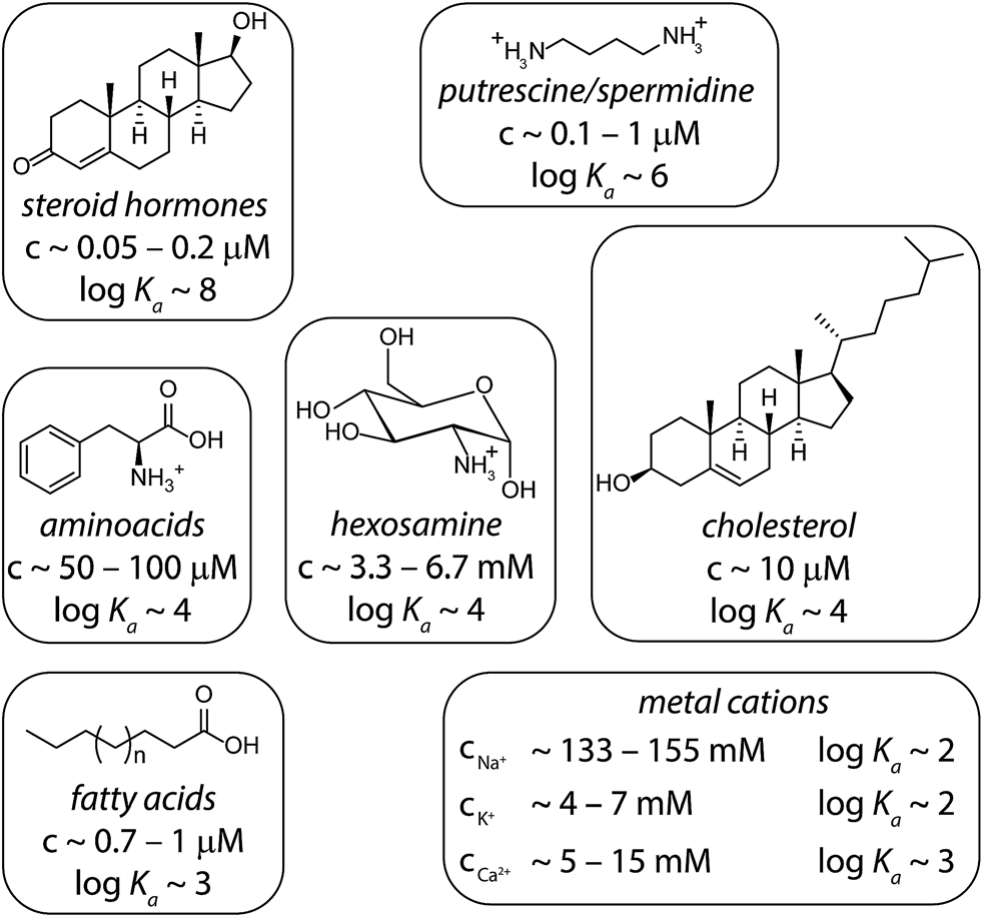
Typical interferents for competitive interactions with CB8 in blood serum. The concentration ranges are depicted as they typically appear in serum.^51^ The logarithmic binding constants are approximated values derived from literature values: steroids,^35^ putrescine/spermidine,^3^,^58^ amino acids,^33^,^65^ hexosamines,^66^ cholesterol,^35^ fatty acids,^67^,^68^ and metal cations.^3^,^69^ Intermolecular interaction parameters for CB*n* complexes can be found in the SupraBank repository.^70^

In order to simulate matrix effects in a complex medium such as blood, a pure competitive behavior of the interferents was assumed. The binding affinities of compounds in blood serum potentially interacting with CB8 were estimated by a critical review of the literature (Fig. 6). The lower and upper limits of blood serum were approximated to be similar to the ranges known for serum.^51^

The competitive model was developed on the basis of a non-linear algebraic system composed of thermodynamic expressions of the equilibrium constants of the individual interferents and the reporter dye with the host CB8 derived from their law of mass action, the corresponding conservations of mass equations. Eqn (8)–(10) describe the algebraic system to be solved. The system was numerically solved to [HD] as signal expression using the software Wolfram Mathematica. For the simulations displayed in this work, the binding constant of the HD complex was varied for each individual IDA titration with increasing **Mem** concentration.8
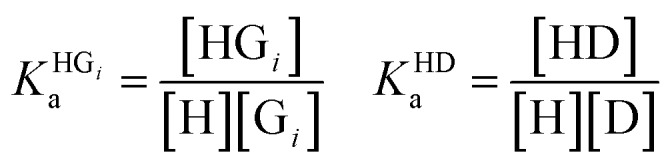

9 [G_*i*_]_0_ = [G_*i*_] + [HG_*i*_] [D]_0_ = [D] + [HD]

10
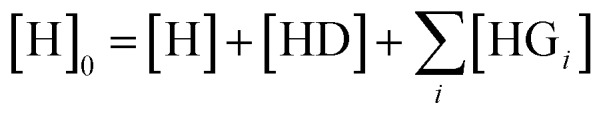



## Conclusions

In this study, we provided the first proof-of-principle report for the *in situ* detection and quantification of Alzheimer's drug memantine in blood serum through an emission-based supramolecular indicator-displacement assay (IDA). We showed that a high-affinity but unselective host, such as cucurbit[8]uril (CB8), cannot be used in a direct-binding assay because of nearly complete, undesirable complex saturation with the naturally occurring interferents in blood serum. Conversely, unselective binding hosts can be utilized for sensing applications in complex, competitively binding biofluids through the IDA. However, this is only possible if both the affinity of the analyte of interest is higher than that of the interferents, and if suitable reporter dyes with a narrowly defined binding affinity range are available. Previously reported indicator dyes for CB8 were not suitable for sensing application in blood serum because they fall short in binding strength, causing high susceptibility to naturally occurring sample-to-sample variations in the blood compositions.

We have introduced here a new class of ultra-high-affinity, emissive indicator dyes for CB8 which is based on a [2.2]paracyclophane moiety that is connected to a pyridinium group. This design ensured ultra-strong binding to CB8 (log *K*_a_ = 12.59 in water) and provided an environment-polarity sensitive CT-type emission signal read out with a desirable, very high Stokes shift (*ca.* 200 nm). With this new dye class, a CB8-based chemosensing ensemble for sensing applications at physiologically relevant memantine concentrations in the chromophoric and autoemissive blood serum could be developed. This is to our knowledge the first working example of a cucurbit[*n*]uril-based sensing assay in blood serum, and ranges amongst the most analyte-concentration sensitive assays reported to date that are based on purely synthetic hosts. We believe that similar concepts can be successfully applied for designing and developing blood-serum based supramolecular assays starting from emerging synthetic high-affinity supramolecular host systems.^71^–^77^

## Conflicts of interest

There are no conflicts to declare.

## Supplementary Material

Supplementary information

Infographic
