# Supplementary material for: Rational design and implementation of a cucurbit[8]uril-based indicator-displacement assay for application in blood serum
Source: Chem Sci. 2019 Jun 4;10(27):6584–93. doi: 10.1039/c9sc00705a (PMC6628674; doi:10.1039/c9sc00705a)
Supplement: Supplementary file 1 [file SC-010-C9SC00705A-s001.pdf]

Electronic Supplementary Material (ESI) for Chemical Science.
This journal is © The Royal Society of Chemistry 2019

Rational Design and Implementation of a Cucurbit[8]uril-based Indicator-Displacement Assay for Application in Blood Serum

Stephan Sinn,[a] Eduard Spuling,[b] Stefan Bräse,[b,c] and Frank Biedermann[a]

a Dr. S. Sinn, Dr. F. Biedermann
Karlsruhe Institute of Technology (KIT), Institute of Nanotechnology (INT),
Hermann-von-Helmholtz-Platz 1, 76344 Eggenstein-Leopoldshafen, (Germany)
E-Mail: frank.biedermann@kit.edu

b E. Spuling, Prof. S. Bräse
Karlsruhe Institute of Technology (KIT), Institute of Organic Chemistry,
Fritz-Haber-Weg 6, 76131 Karlsruhe (Germany)

c Prof. S. Bräse
Karlsruhe Institute of Technology (KIT)
Institute of Toxicology and Genetics (ITG)
Hermann-von-Helmholtz-Platz 1
76344 Eggenstein-Leopoldshafen, (Germany).

Email correspondence should be directed to frank.biedermann@kit.edu

Table of Contents

1. General Information 2
1.1. Abbreviations 2
2. Simulations 2
3. Experimental procedures 9
3.1. Synthesis of the indicator dye MPCP 9
3.1.1. Synthesis of (rac)-4-bromo[2.2]paracyclophane (BrPCP) 9
3.1.2. Synthesis of (rac)-4-pyridyl[2.2]paracyclophane (PyPCP) 10
3.1.3. Synthesis of (rac)-*N*-methyl-4-pyridinylium[2.2]paracyclophane (MPCP) 10
3.2. Photophysical characterization of the indicator dye MPCP 12
3.3. Host-guest inclusion complex formation of MPCP with CB8 15
3.3.1. DFT calculations 15
3.3.2. Mass spectrometry 16
3.3.3. NMR spectroscopy 17
3.3.4. Preparation of stock solutions for photophysical host-guest binding experiments 17
3.3.5. UV-Vis absorption spectroscopy 18
3.3.6. Fluorescence spectroscopy 20
3.3.7. Binding isotherms 22
3.3.8. Indicator displacement assay in blood serum 25
3.4. Host-guest inclusion complex formation of MPCP with CB7 28
3.4.1. DFT calculations 28
3.4.2. NMR spectroscopy 29
3.5. ^{1}H and ^{13}C NMR spectra of the MPCP precursors and of MPCP 30
4. References 33

1. General Information

1.1. Abbreviations

H	host
G	guest (= analyte, interferant)
D	indicator dye
Mem	memantine
BC	berberine chloride
CB8	cucurbit[8]uril
CB7	cucurbit[7]uril
BrPCP	(*rac*)-4-bromo[2.2]paracyclophane
PyPCP	(*rac*)-4-pyridyl[2.2]paracyclophane
MPCP	(*rac*)-*N*-methyl-4-pyridinylium[2.2]paracyclophane
DCM	dichloromethane
MeCN	acetonitrile
EtAc	ethyl acetate
EtOH	ethanol
MeOH	methanol
NaAc	sodium acetate buffer
PBS	phosphate buffered saline
HPLC	high pressure liquid chromatography
MS	mass spectrometry
NMR	nuclear magnetic resonance
λ	wavelength
ε	molar extinction coefficient
I_{em}	emission intensity

2. Simulations

In order to simulate matrix effects in a complex medium such as blood, a pure competitive behavior of the interferants was assumed. The binding affinities of compounds in blood serum that can interact with CB8 were estimated by a critical review of the literature (see main text for details).

The direct binding assay was developed on the basis of a non-linear algebraic system composed of thermodynamic expressions of the equilibrium constants of the individual interferants and the target analyte Mem with the host CB8. The equations were derived from their law of mass action and the corresponding conservations of mass. Eqs. S 1 – S 3 describe the algebraic system to be solved. For the simulations displayed in this work, the binding constant of the H⊃Mem complex was fixed at log K_a = 12.92 as was determined in this work, see main text. The simulations were carried out for 5 µM host (H). The Mem concentration was varied from 0 to 5 µM. See the main text and Fig. 6 for the

interferants present in blood serum and their concentration ranges. The system was numerically solved to [H] as signal expression (fluorescent host implemented) using the software Wolfram Mathematica. The increasing concentration (abscise) during a titration is displayed as its initial concentration marked with a subscript 0. The equilibrium concentrations are displayed without this subscription.

$$K_a^{HG_i} = \frac{[\mathrm{HG}_i]}{[\mathrm{H}] \cdot [G_i]} \quad \text{Eq. S 1}$$

$$[G_i]_0 = [G_i] + [HG_i] \quad \text{Eq. S 2}$$

$$[H]_0 = [H] + \sum_i [HG_i] \quad \text{Eq. S 3}$$

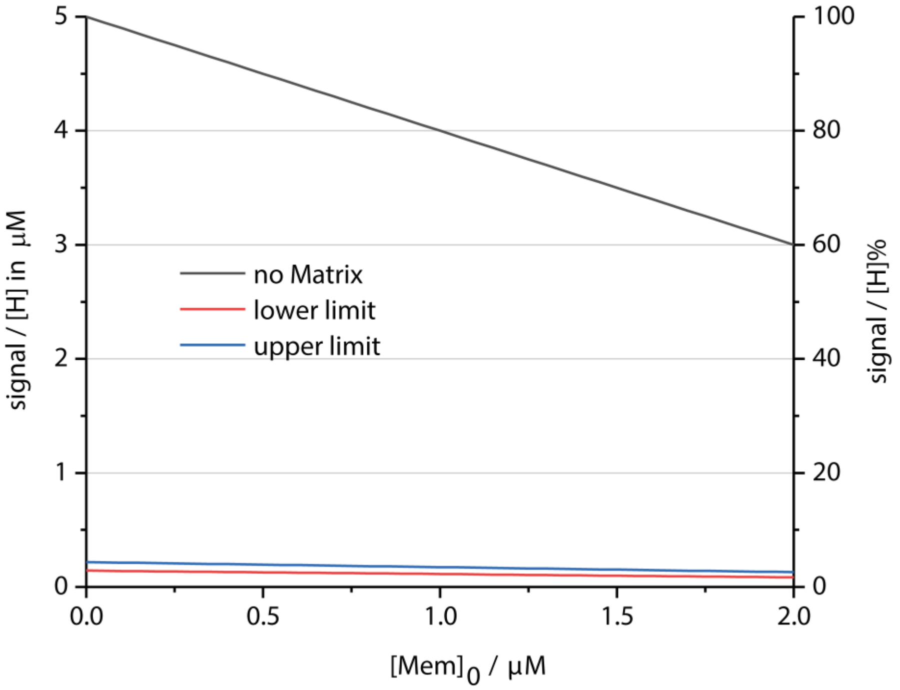

Fig. S 1: Simulations of the effect of blood matrix interferants on the CB8 direct binding assay performance, comparing the lowest limit of interference concentration (red curve) and the highest limit (blue curve) for blood serum matrices and no matrix effects (black curve).

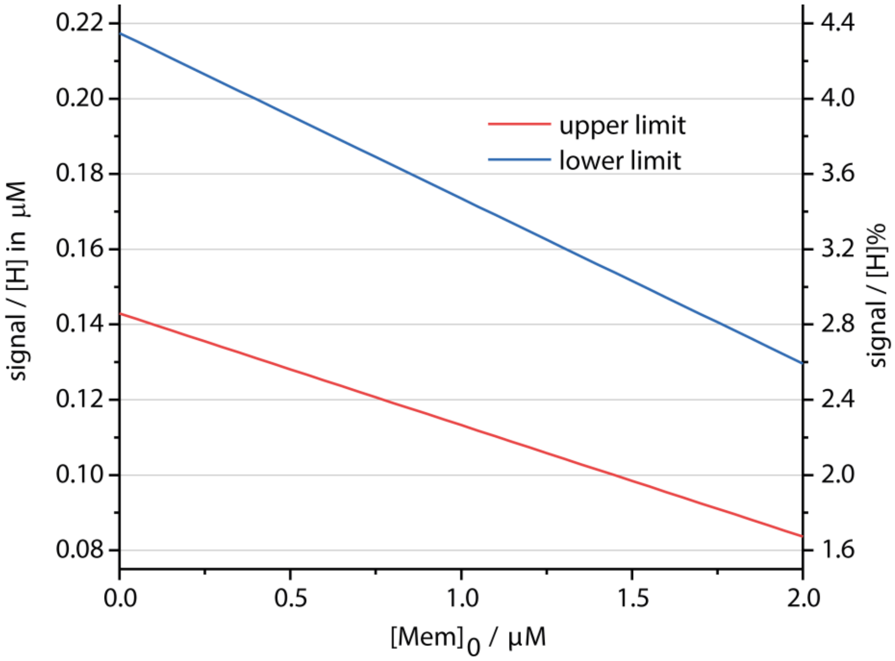

Fig. S 2: Simulations (zoom-in) of the effect of blood matrix interferants on the CB8 direct binding assay performance, comparing the lowest limit of interference concentration (red curve) and the highest limit (blue curve) for blood serum matrices. See the main text for the interferants present in blood serum and their concentration ranges.

The competitive model was developed on the basis of a non-linear algebraic system composed of thermodynamic expression of the equilibrium constants of the individual interferants, the target analyte Mem and the reporter dye with the host CB8. The equations were derived from their law of mass action and the corresponding conservations of mass. Eqs. S 4 – S 6 describe the algebraic system to be solved. The binding constant of the H⊃D complex was fixed for each IDA titration with increasing Mem concentration (up to 5 µM). For the simulations displayed in this work, the binding constant of the H⊃Mem was fixed at log K_a = 12.92 as was determined in this work, see main text. The simulations were carried out for 5 µM host (H) and indicator dye (D). See the main text and Fig. 6 for the interferants present in blood serum and their concentration ranges. The system was numerically solved to [HD] as signal expression (fluorescent host-dye complex) using the software Wolfram Mathematica. The increasing concentration (abscise) during a titration is displayed as its initial concentration marked with a subscript 0. The equilibrium concentrations are displayed without this subscription.

$$K_a^{HG_i} = \frac{[\mathrm{HG}_i]}{[\mathrm{H}] \cdot [G_i]} \quad K_a^{HD} = \frac{[\mathrm{H}D]}{[\mathrm{H}] \cdot [D]}$$ Eq. S 4

$$[G_i]_0 = [G_i] + [HG_i] \quad [D]_0 = [D] + [HD]$$ Eq. S 5

$$[H]_0 = [H] + [HD] + \sum_i [HG_i]$$ Eq. S 6

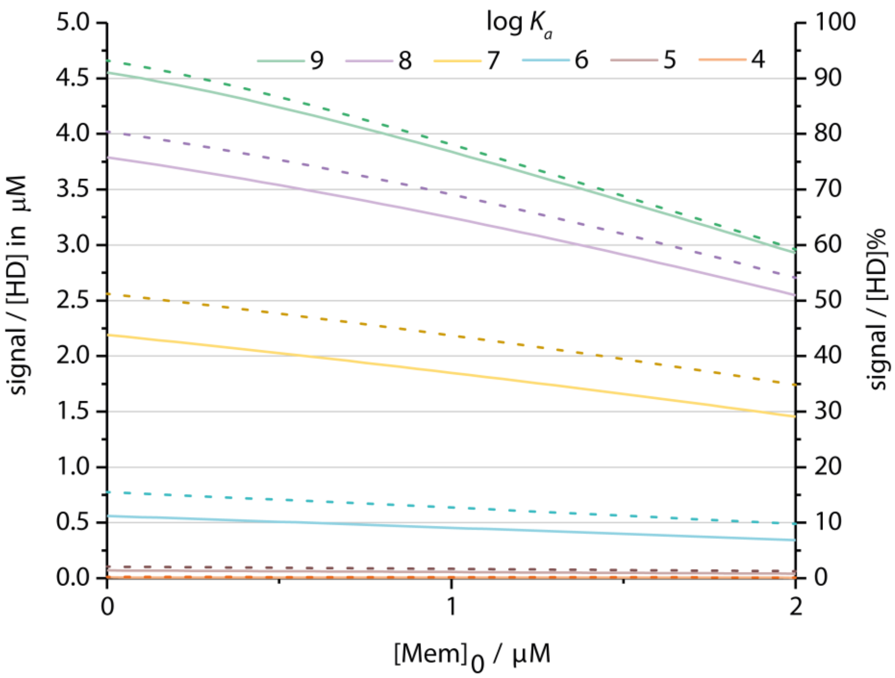

Fig. S 3: Simulations of the effect of blood matrix interferants on the CB8-memantine IDA assay performance, comparing the lowest limit of interference concentration (dotted curves) and the highest limit (solid curves) for blood serum matrices. Indicator dyes with log K_a ≤9 for CB8 were considered. See the main text for the interferants present in blood serum and their concentration ranges.

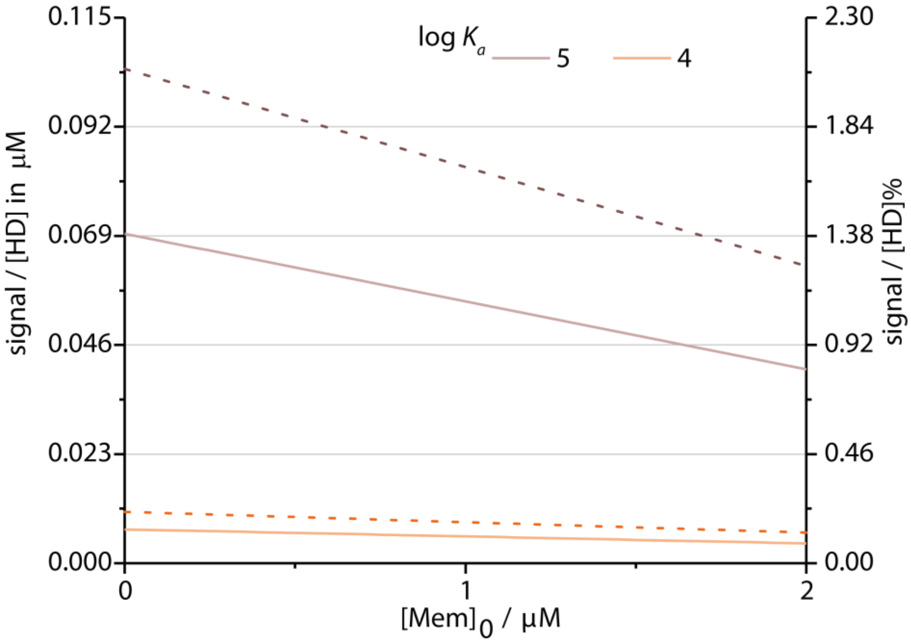

Fig. S 4: Simulations (zoom-in) of the effect of blood matrix interferants on the CB8-memantine IDA assay performance, comparing the lowest limit of interference concentration (dotted curves) and the highest limit (solid curves) for blood serum matrices. Indicator dyes with log K_a 4 and 5 for CB8 were considered. See the main text for the interferants present in blood serum and their concentration ranges.

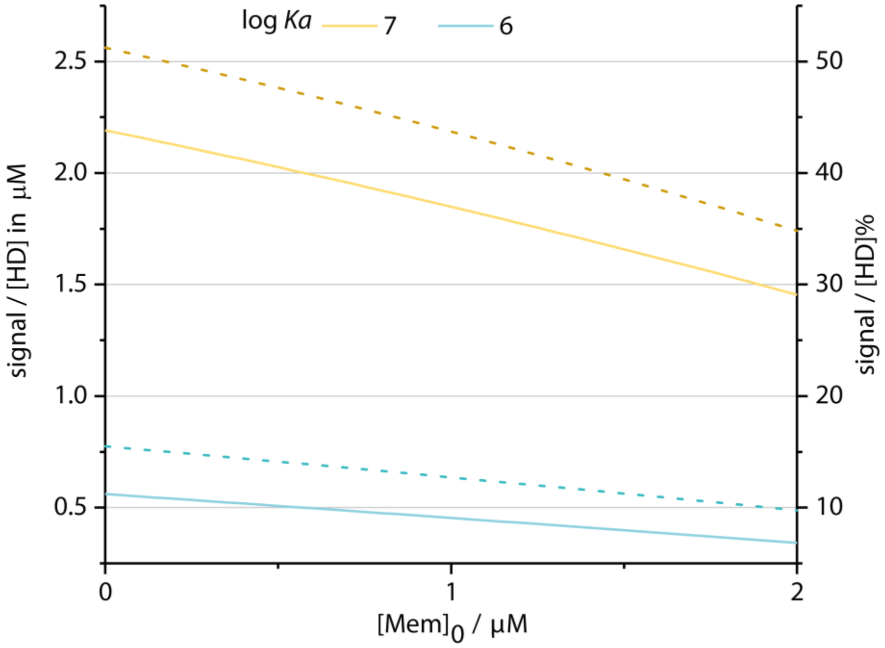

Fig. S 5: Simulations (zoom-in) of the effect of blood matrix interferants on the CB8-memantine IDA assay performance, comparing the lowest limit of interference concentration (dotted curves) and the highest limit (solid curves) for blood serum matrices. Results for indicator dyes with log K_a 7 and 6 for CB8 are shown. See the main text for the interferants present in blood serum and their concentration ranges.

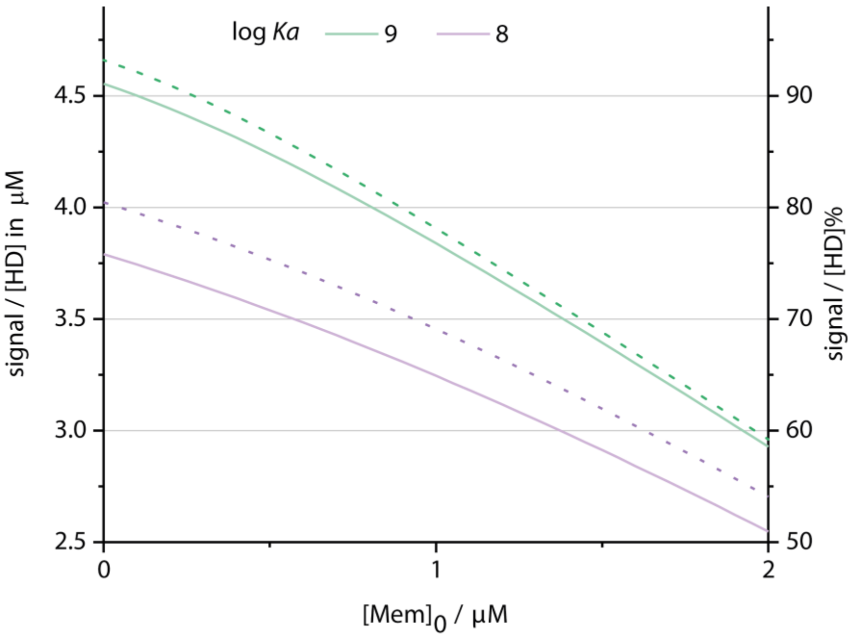

Fig. S 6: Simulations (zoom-in) of the effect of blood matrix interferants on the CB8-memantine IDA assay performance, comparing the lowest limit of interference concentration (dotted curves) and the highest limit (solid curves) for blood serum matrices. Indicator dyes with log K_a 8 and 9 for CB8 were considered. See the main text for the interferants present in blood serum and their concentration ranges.

The simulation revealed that the curves at lower and higher concentration of the blood serum matrix interferents are equal when a high-affinity dye (log $K_a \geq 11$) is present (Fig. S 7 to S 10).

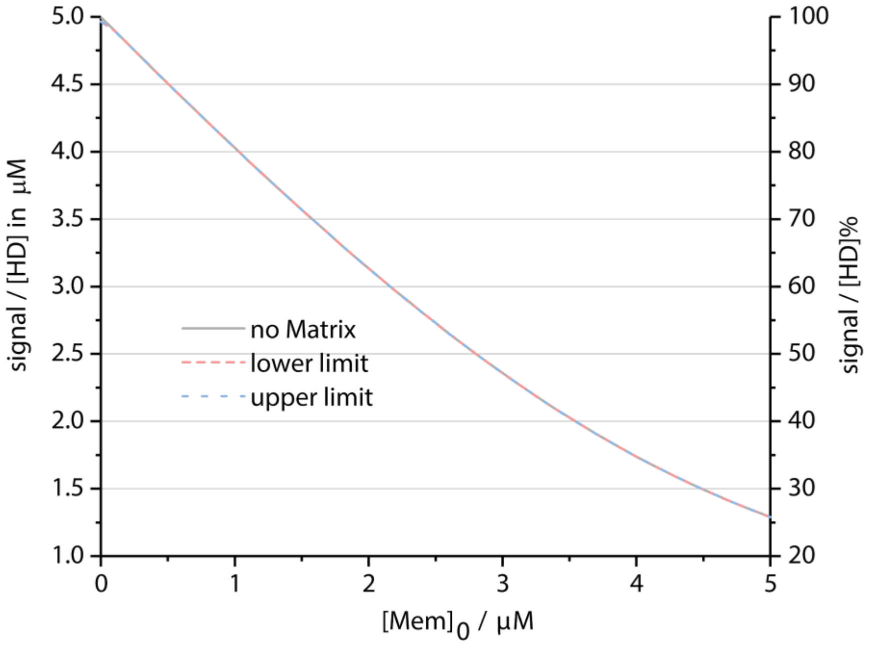

Fig. S 7: Simulations of the effect of blood matrix interferants on the CB8-memantine IDA assay performance, comparing the lower limit of interference concentration (dotted red curves) and the upper limit (dashed blue curve) for blood serum matrices and matrix free IDA (black solid curve). All three curves overlay. The log K_a of CB8⊃indicator was set to be 12. See the main text for the interferants present in blood serum and their concentration ranges.

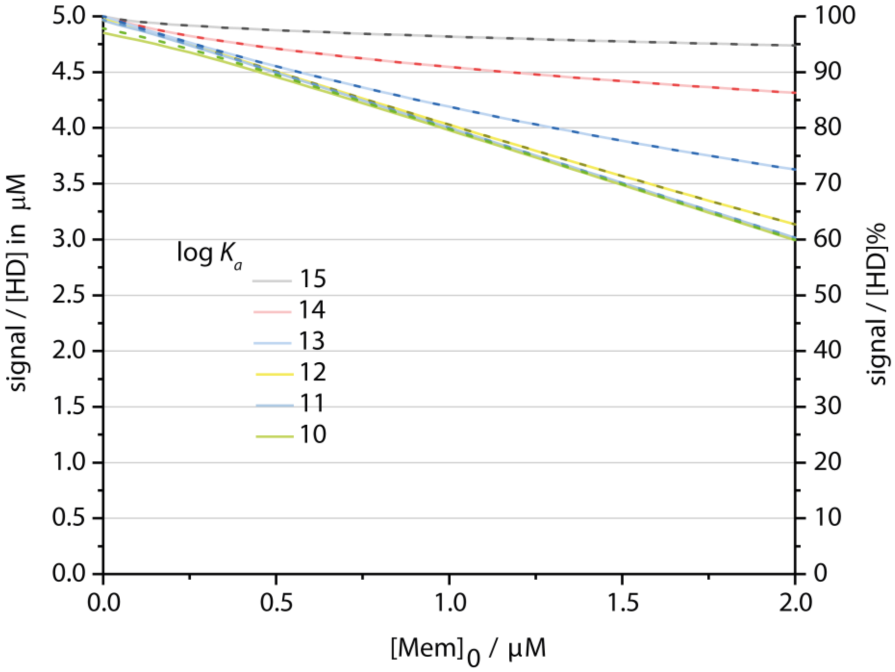

Fig. S 8: Simulations of the effect of blood matrix interferants on the CB8-memantine IDA assay performance, comparing the lowest limit of interference concentration (dotted curves) and the highest limit (solid curves) for blood serum matrices. Indicator dyes with log K_a ≥10 for CB8 were considered. See the main text for the interferants present in blood serum and their concentration ranges.

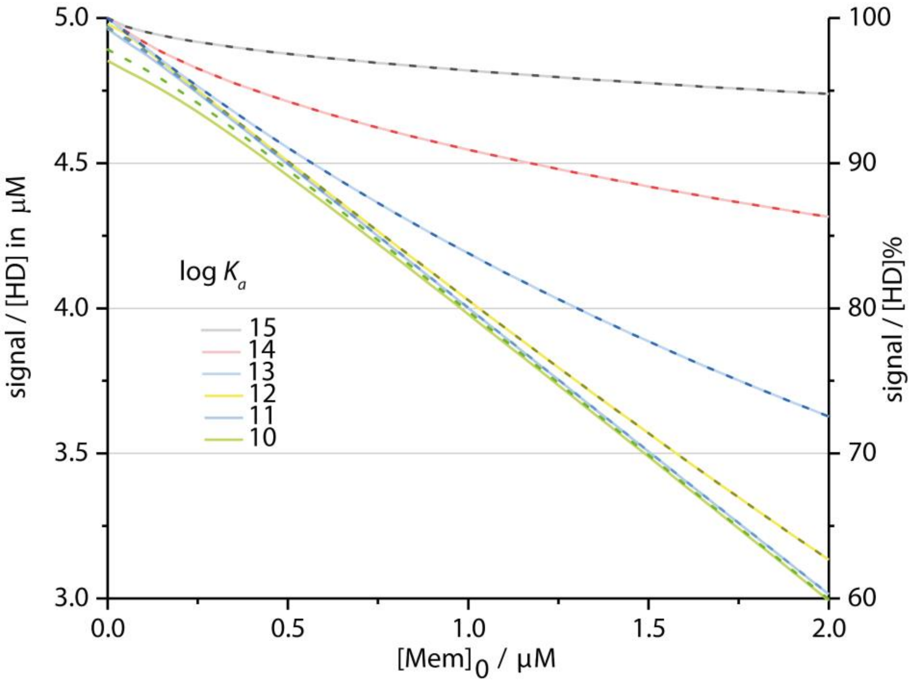

Fig. S 9: Simulations (zoom-in of the effect of blood matrix interferants on the CB8-memantine IDA assay performance, comparing the lowest limit of interference concentration (dotted curves) and the highest limit (solid curves) for blood serum matrices. Indicator dyes with log K_a ≥10 for CB8 were considered. See the main text for the interferants present in blood serum and their concentration ranges.

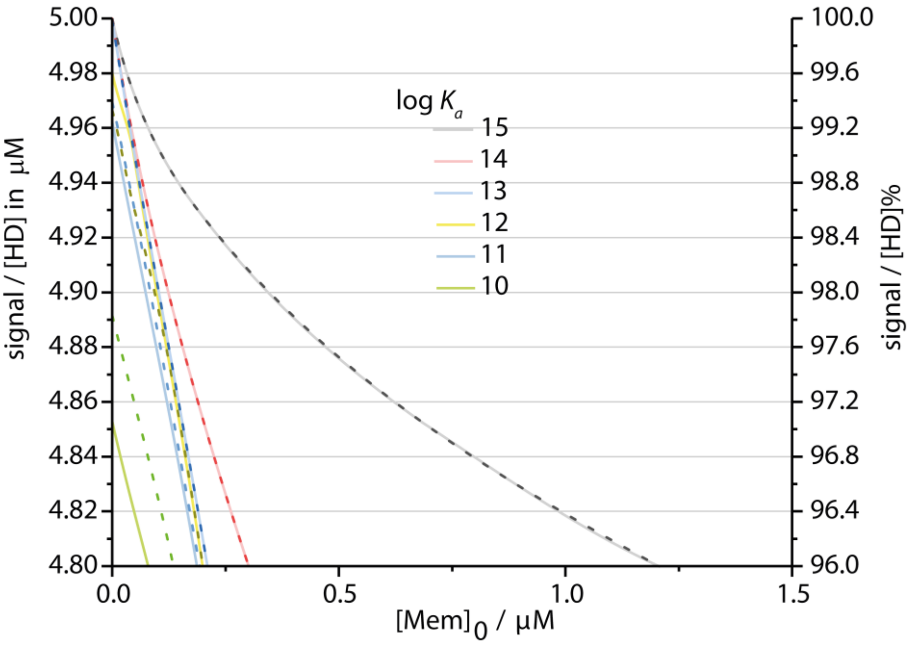

Fig. S 10: Simulations (zoom-in) of the effect of blood matrix interferants on the CB8-memantine IDA assay performance, comparing the lowest limit of interference concentration (dotted curves) and the highest limit (solid curves) for blood serum matrices. Indicator dyes with log K_a ≥10 for CB8 were considered. See the main text for the interferants present in blood serum and their concentration ranges.

3. Experimental procedures

3.1. Synthesis of the indicator dye MPCP

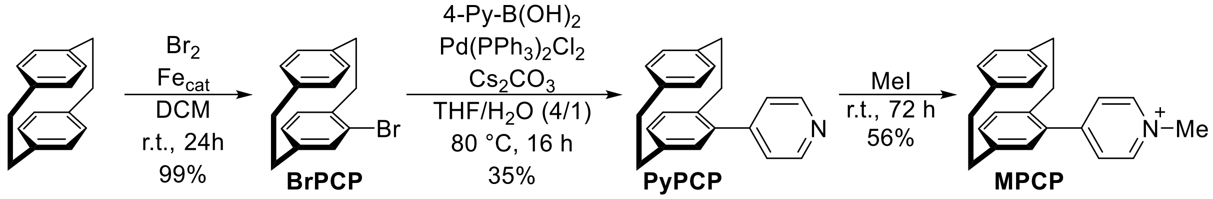

Scheme S 1: Synthesis of the indicator **MPCP** from the commercial [2.2]paracyclophane conducted for this investigation.

3.1.1. Synthesis of (rac)-4-bromo[2.2]paracyclophane (BrPCP)

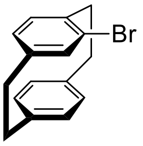

To 80.3 mg iron powder (1.44 mmol, 2.00 mol%) were added 5 mL of a solution of 3.77 mL bromine in 80 mL (11.7 g, 73.2 mmol, 1.02 equiv.) dichloromethane. After stirring for 1 h, the reaction mixture was diluted with 250 mL dichloromethane and 15.0 g [2.2]paracyclophane (72.0 mmol, 1.00 equiv.) were added. The mixture was stirred for further 20 min, followed by dropwise addition of the residual bromine solution over 5 h. The reaction mixture was stirred overnight and a saturated aqueous solution of $Na_2S_2O_3$ was added. The aqueous phase was extracted with dichloromethane (2 × 50 mL) and the combined organic layers were dried over Na_2SO_4. The solvent was removed under reduced pressure to obtain **BrPCP** (20.5 g, 71.4 mmol, 99%) as a colorless solid without further purification.

m.p. 250 °C, R_f = 0.77 (cyclohexane/ethyl acetate = 7:1). – ^{1}H NMR (400 MHz, $CDCl_3$): δ = 7.17 (dd, *J* = 7.8, 1.9 Hz, 1H, H_{Ar}), 6.57 (dd, *J* = 7.9, 1.7 Hz, 1H, H_{Ar}), 6.56–6.48 (m, 5H, H_{Ar}), 3.52–3.42 (m, 1H, H_{Pc}), 3.26–3.06 (m, 5H, H_{Pc}), 2.96–2.80 (m, 2H, H_{Pc}) ppm. – ^{13}C NMR (100 MHz, $CDCl_3$): δ = 141.7 (C_q, C_{Ar}), 139.4 (C_q, C_{Ar}), 139.2 (C_q, C_{Ar}), 137.4 (+, CH, C_{Ar}), 135.1 (+, CH, C_{Ar}), 133.3 (+, CH, C_{Ar}), 133.1 (C_q, C_{Ar}), 133.0 (+, CH, C_{Ar}), 132.3 (+, CH, C_{Ar}), 131.5 (+, CH, C_{Ar}), 128.5 (+, CH, C_{Ar}), 127.1 (C_q, C_{Ar}), 36.0 (–, CH_2), 35.6 (–, CH_2), 34.9 (–, CH_2), 33.6 (–, CH_2) ppm. – IR (ATR): $\tilde{\nu}$ = 3007 (vw), 2924 (w), 2887 (w), 2849 (w), 1893 (vw), 1585 (w), 1542 (w), 1512 (vw), 1497 (w), 1476 (w), 1448 (vw), 1431 (w), 1409 (w), 1390 (w), 1319 (vw), 1185 (w), 1092 (vw), 1034 (w), 941 (vw), 897 (w), 839 (w), 792 (w), 708 (w), 667 (w), 640 (w), 576 (w), 513 (m), 472 (w), 404 (vw) cm^{-1}. – MS (70 eV, EI), *m/z* (%): 288/286 (19/20) $[M]^+$, 184/182 (13/13) $[M– C_8H_8]^+$, 104 (100) $[C_8H_8]^+$. – HRMS ($C_{16}H_{15}Br$) calc.: 286.0352; found: 286.0352. The analytical and spectral properties match those reported in the literature.[1] See **Fig. S 34** and **Fig. S 35** for the ^{1}H and ^{13}C NMR spectra of the compound.

3.1.2. Synthesis of (rac)-4-pyridyl[2.2]paracyclophane (PyPCP)

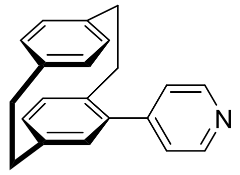

The coupling of 4-pyridylboronic acid with 4-bromo[2.2]paracyclophane **BrPCP** was performed according to a literature known procedure optimized for this work.[2] *rac*-4-Bromo[2.2]paracyclophane (**BrPCP**) (201 mg, 700 µmol, 1.00 equiv.), 4-pyridylboronic acid (112 mg, 910 µmol, 1.30 equiv.), Cs_2CO_3 (342 mg, 1.05 mmol, 1.50 equiv.) and $Pd(PPh_3)_2Cl_2$ (28.1 mg, 40.0 µmol, 6 mol%) were sealed in a pressure tube, which was first evacuated then flushed with argon 3 times. Through the septum 3 mL of tetrahydrofurane/water (4:1) was added, heated to 80 °C and stirred for 16 h. The reaction mixture was diluted with water (20 mL) and extracted with ethyl acetate (3 x 25 mL). The combined organic layers were dried over Na_2SO_4, filtered and concentrated under reduced pressure to remove the solvents. The residue war purified by column chromatography (silica gel, cyclohexane/ethyl acetate = 2:1) to give *(rac)*-4-pyridyl[2.2]paracyclophane (**PyPCP**) as a white solid (70.0 mg, 245 µmol 35%).

M.p. 115–118 °C. – R_f (cyclohexane/ethyl acetate = 2:1) = 0.43. – ^{1}H NMR (400 MHz, $CDCl_3$): δ = 8.70 (d, *J* = 4.8 Hz, 2H), 7.40 (d, *J* = 4.8 Hz, 2H), 6.65 – 6.62 (m, 2H), 6.59 – 6.56 (m, 3H), 6.54 (s, 2H), 3.44 – 3.37 (m, 1H), 3.21 – 3.12 (m, 3H), 3.09 – 2.87 (m, 3H), 2.69 – 2.63 (m, 1H) ppm. – ^{13}C NMR (100 MHz, $CDCl_3$): δ = 150.2 (+,CH), 148.7 ($C_{q.}$), 140.3 ($C_{q.}$), 139.7 ($C_{q.}$), 139.3 ($C_{q.}$), 137.4 ($C_{q.}$), 136.3 (+, CH), 133.6 (+, CH), 133.4 (+, CH), 132.9 (+, CH), 132.2 (+, CH), 132.1 (+, CH), 129.9 (+, CH), 124.6 (+, CH), 35.6 (–, CH_2), 35.4 (–, CH_2), 35.1 (–, CH_2), 34.1 (–, CH_2) ppm. – IR (ATR) ῡ = 2925 (w), 2850 (w), 1895 (w), 1594 (w), 1497 (w), 1474 (vw), 1400 (w), 1211 (vw), 1093 (w), 1066 (vw), 991 (w), 939 (vw), 905 (w), 850 (w), 825 (w), 716 (w), 667 (vw), 646 (w), 622 (w), 594 (w), 563 (w), 515 (w), 483 (w), 423 (vw) cm^{-1}. – MS (EI, 70 eV) m/z (%) = 286 (25) $[M+H]^+$, 285 (100) $[M]^+$, 181 (62) $[M–C_8H_7]$+, 180 (100) $[M–C_8H_8]^+$, 105 (51) $[C_8H_9]^+$, 104 (61) $[C_8H_8]^+$. – HRMS ($C_{21}H_{19}N$) calc. 285.1512, found 285.1511 See **Fig. S 36** and **Fig. S 37** for the ^{1}H and ^{13}C NMR spectra of the compound.

3.1.3. Synthesis of (rac)-*N*-methyl-4-pyridinylium[2.2]paracyclophane (MPCP)

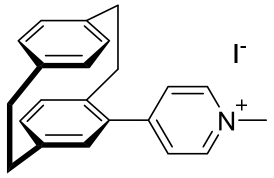

PyPCP (50.0 mg, 180 µmol, 1.00 equiv.) was dissolved in 1.5 mL DCM in a one neck round bottom flask equipped with a magnetic stirrer. Subsequently, 1 mL of MeI (2.28 g, 16.1 mmol, 894 equiv.) was added at ambient conditions resulting in pale yellow solution. The crude mixture was allowed to react for 72 h, while two portions of MeI (1 mL) were additionally added after 24 h and 48 h. The crude reaction mixture was extracted three times with water. After the removal of water by lyophilization, **MPCP** was obtained as a yellow powder (42.1 mg, 98.5 µmol, 54.7%). ^{1}H NMR (500 MHz, D_2O) δ = 8.76 (d, *J*=6.4, 2H), 8.14 (d, *J*=6.5, 2H), 6.86 (d, *J*=11.5, 3H), 6.80 – 6.68 (m, 3H), 6.53 (d, *J*=7.9, 1H), 4.38 (s, 3H), 3.45 – 3.35 (m, 1H), 3.25 – 3.06 (m, 5H), 3.03 – 2.94 (m, 1H), 2.62 – 2.52 (m, 1H) ppm. – ^{13}C NMR (126 MHz, D_2O): δ = 144.6 (+, CH), 141.6 ($C_{q.}$), 140.6 ($C_{q.}$), 140.0 ($C_{q.}$), 138.8 ($C_{q.}$),

137.0 (+, CH), 135.9 (+, CH), 135.8 ($C_{q.}$), 133.7 (+, CH), 133.2 (+, CH), 132.4 (+, CH), 131.8 (+, CH), 129.5 (+, CH), 127.7 (+, CH), 47.2 (+, CH_3), 34.6 (–, CH_2), 34.3 (–, CH_2), 34.2 (–, CH_2), 33.4 (–, CH_2) ppm. – IR (ATR) $\tilde{\upsilon}$ = 3386 (vw), 3032 (vw), 2920 (vw), 2850 (vw), 2573, (vw), 2322 (vw), 2168 (vw), 2020 (vw), 1918 (vw), 1639 (vw), 1516 (vw), 1454 (vw), 1332 (vw), 1198 (vw), 1096 (vw), 949 (vw), 843 (w), 720 (vw), 642 (vw), 488 (vw) cm^{-1}. – HRMS (ESI, $C_{22}H_{22}N^+$) calc. 300.1747, found 300.1737. – EA ($C_{22}H_{22}IN{\cdot}5H_2O$) calc. C 51.06%, H 4.29%, N 2.71%; found C 50.90%, H 4.29%, N 2.81%. See **Fig. S 38** and **Fig. S 39** for the ^1H and ^{13}C NMR spectra of the compound.

3.2. Photophysical characterization of the indicator dye MPCP

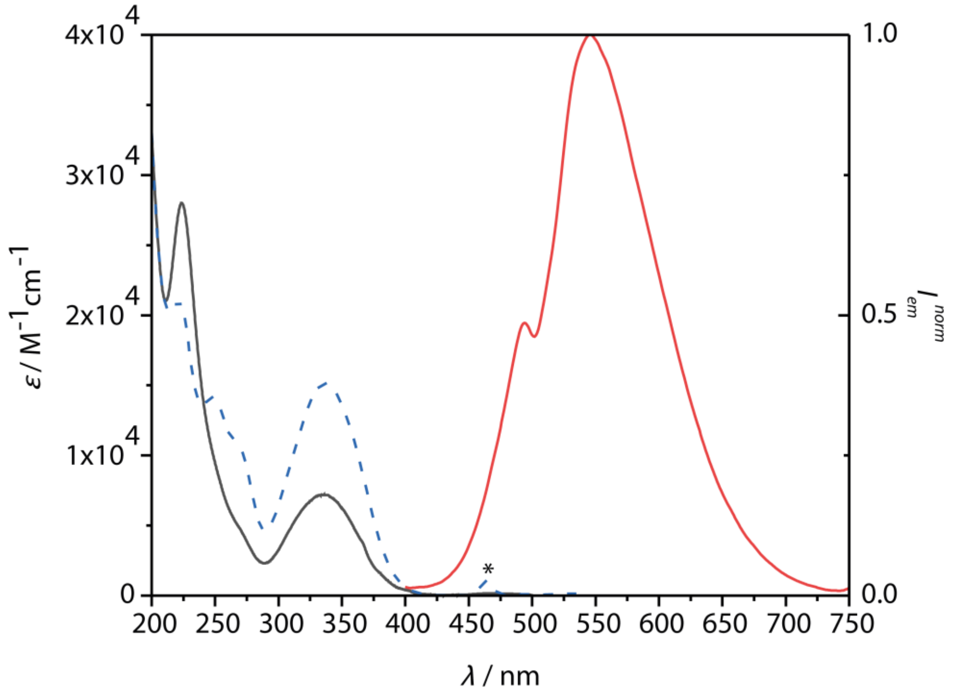

Fig. S 11: Extinction (black, solid, OD units), normalized emission (red, solid, λ_{ex} = 335 nm) and excitation spectra (blue, dashed, arbitrary units, λ_{em} = 550 nm) of an aqueous solution of **MPCP** (c = 1.65 µM). The asterisk indicates an instrumental artefact (Raman scattering band).

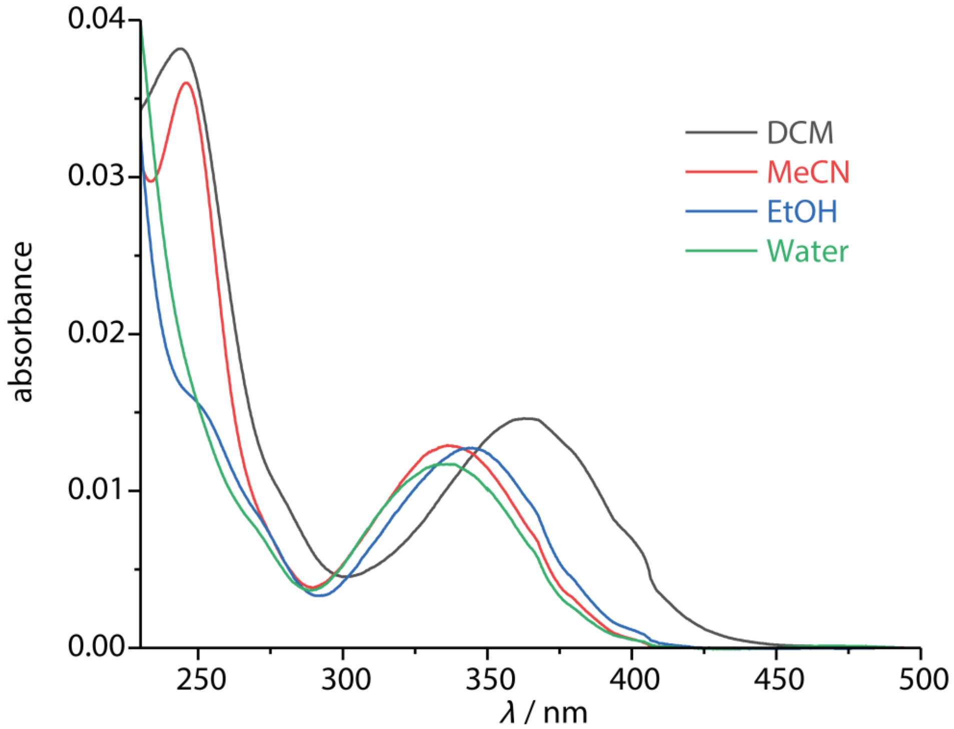

Fig. S 12: UV-Vis absorption spectra of **MPCP** (c = 2 µM) in different solvents, showing the polarity dependence of the CT band around 335 nm (water).

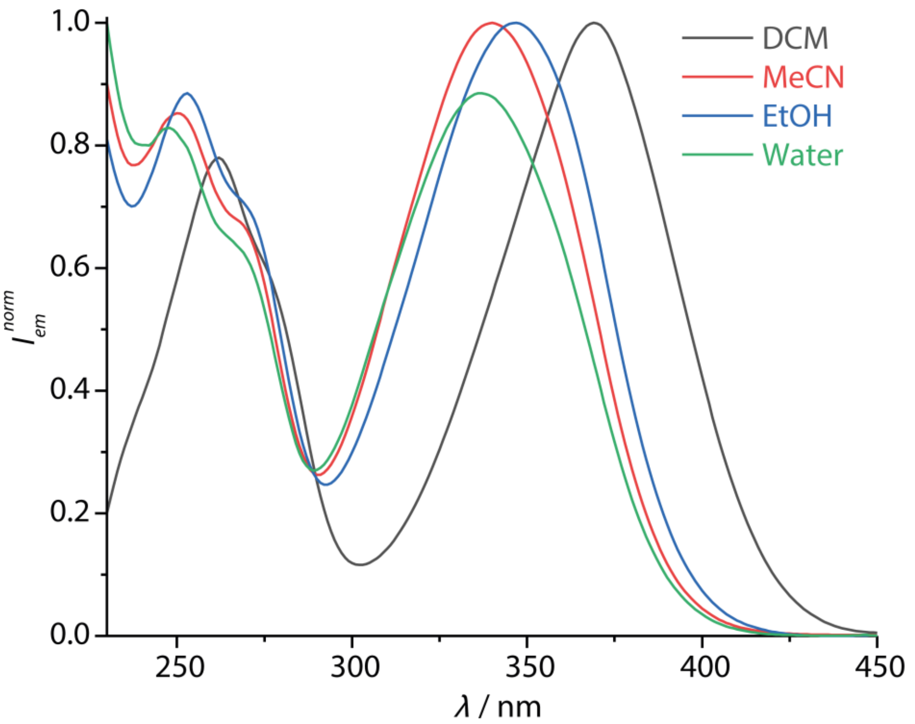

Fig. S 13: Fluorescence excitation spectra (λ_{em} = 550 nm) of **MPCP** (c = 2 µM) in different solvents, showing the polarity dependence of the CT band around 335 nm (water).

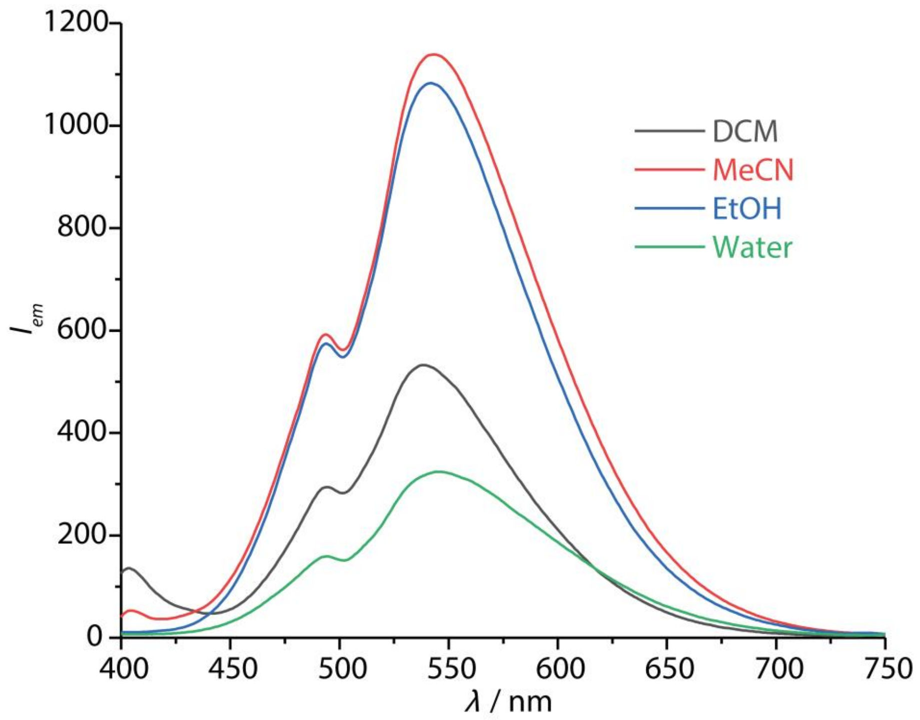

Fig. S 14: Fluorescence emission spectra (λ_{exc} = 335 nm) of **MPCP** (c = 2 µM) in different solvents, showing the polarity dependence of the CT band around 335 nm (water).

Table S 1: Solvent dependence of the ICT band of **MPCP** in electronic absorption spectroscopy

solvent	λ_{ICT} / nm Absorption	λ_{ICT} / nm Fluorescence	$\Delta\tilde{\nu}$ / cm^{-1}	E_T(30) / kJ/mol
**DCM**	363	538	9037	170
**MeCN**	337	543	11257	190
**EtOH**	345	542	10535	217
**Water**	335	545	11502	264

T=25 °C, listed by the solvent polarity from low to high (top to bottom) by using E_T(30) values as reference.[3]

3.3. Host-guest inclusion complex formation of MPCP with CB8

3.3.1. DFT calculations

Density functional theory (DFT) calculations have been carried out in order to evaluate the ground state structure of the inclusion complex between CB8 and **MPCP** as well as CB7 and **MPCP**. DFT calculations were carried out utilizing the hybrid functional B3LYP with the standard valence basis set 6-31G(d,p) for C,H,N and O. The found stationary points possess no imaginary frequencies and therefore assumed to be true minima. All calculations were performed with the program package G16.B.01.[4]

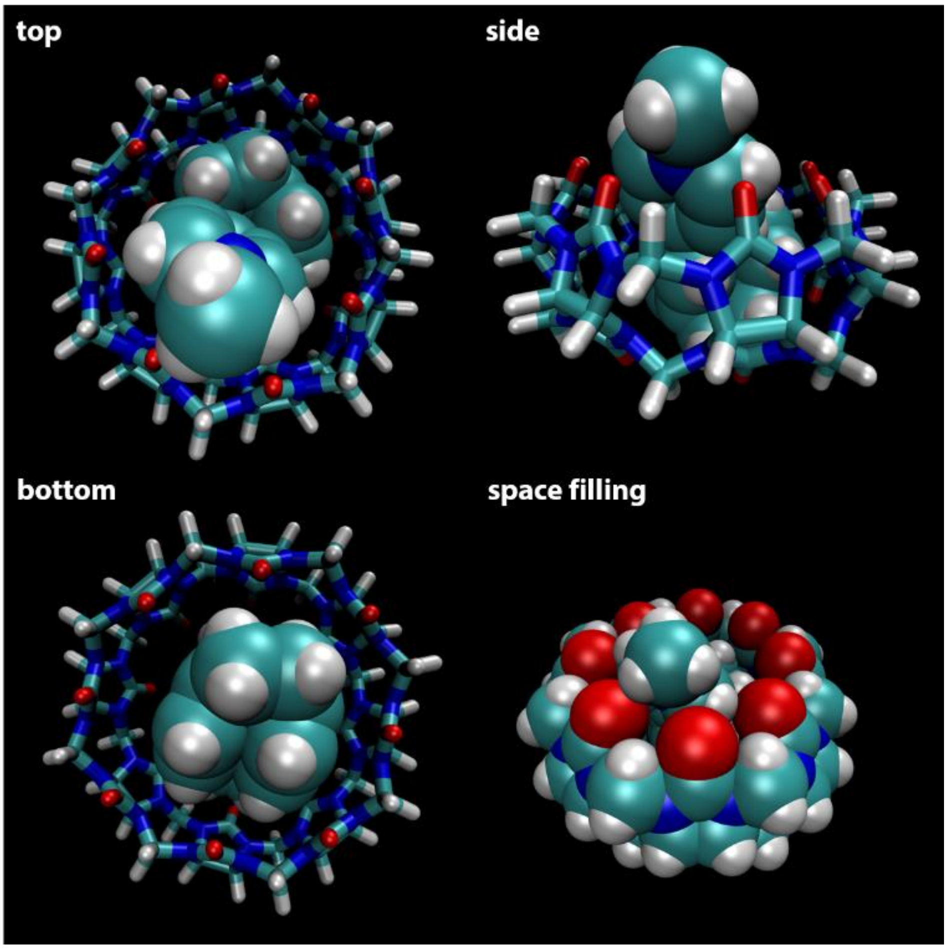

Fig. S 15: DFT ground-state optimized host:guest complex structure of CB8⊃**MPCP** in top, side and bottom view featuring CB8 in licorice and **MPCP** in space filling representation. On the bottom right both entities are displayed in space filling style from a top perspective. DFT calculations were carried out utilizing the hybrid functional B3LYP with the standard valence basis set 6-31G(d,p) for C,H,N and O.

3.3.2. Mass spectrometry

Fig. S 16 and **Fig. S 17** show the ESI-ToF MS spectra of **MPCP** and CB8⊃**MPCP** indicating the formation of the supramolecular complex. The entire ESI-ToF-MS spectrum lacks the presence of peaks indicating other complexes favoring a different constitution than the 1:1 binding model.

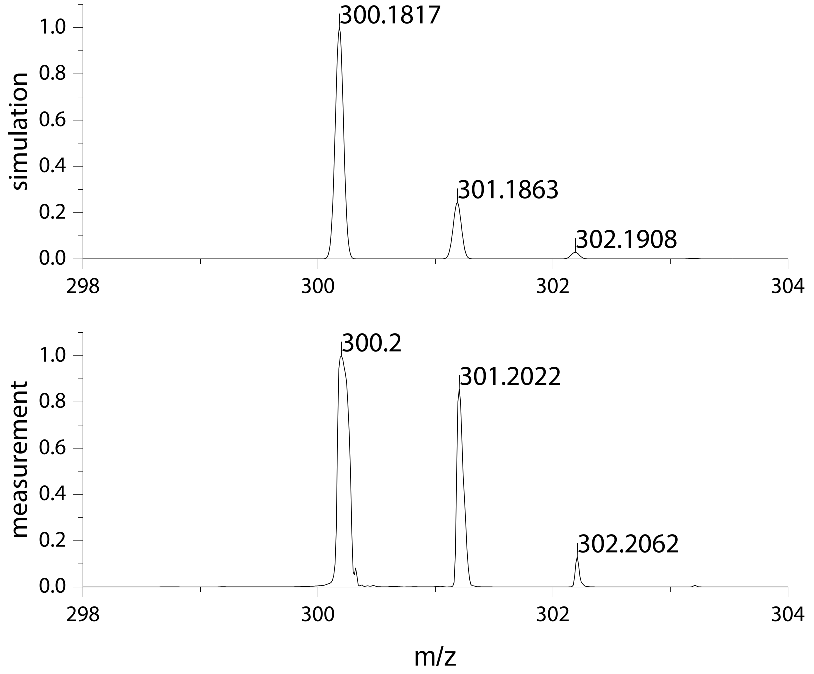

Fig. S 16: Simulated (top) and measured (bottom) ESI-ToF-MS spectrum of **MPCP**.

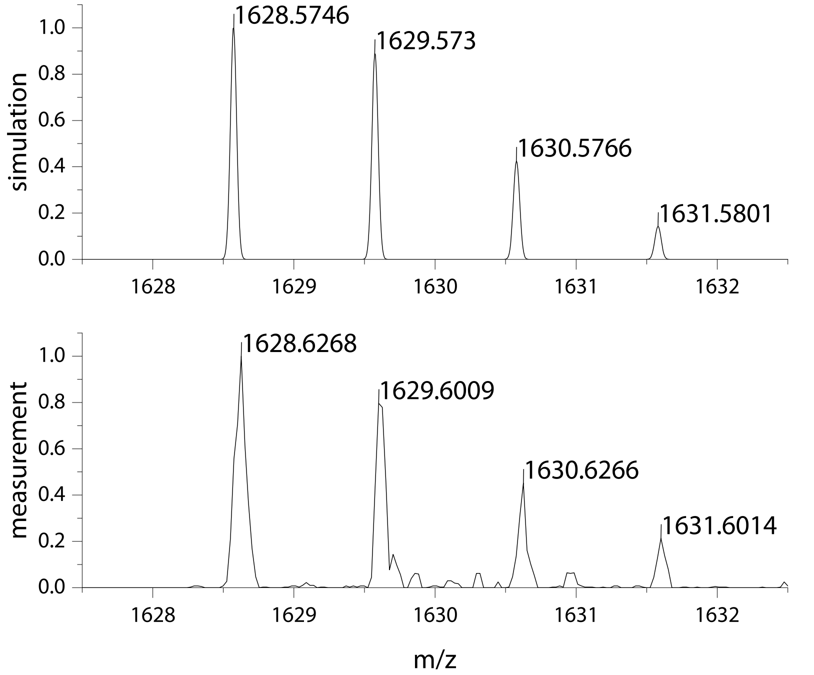

Fig. S 17: Simulated (top) and measured (bottom) ESI-ToF-MS spectrum of CB8⊃**MPCP**.

3.3.3. NMR spectroscopy

NMR experiments with **MPCP** in deuterium oxide allow the assignment of all present protons and are in agreement with recorded NMR spectra of the parent PCP species (**Fig. S 38**). Addition of CB8 to a **MPCP** solution in D_2O (1:1 molar ratio) is accompanied by a high field shift of the **MPCP** proton signals indicating the formation of an inclusion complex (**Fig. S 18**, blue curve). The chemical shifts of the **MPCP** protons remain constant when the concentration of CB8 (**Fig. S 18**, red curve) is raised to a large molar excess in comparison to **MPCP**. In addition to the results of MS experiments (*vide supra*) a 1:1 binding model is concluded.

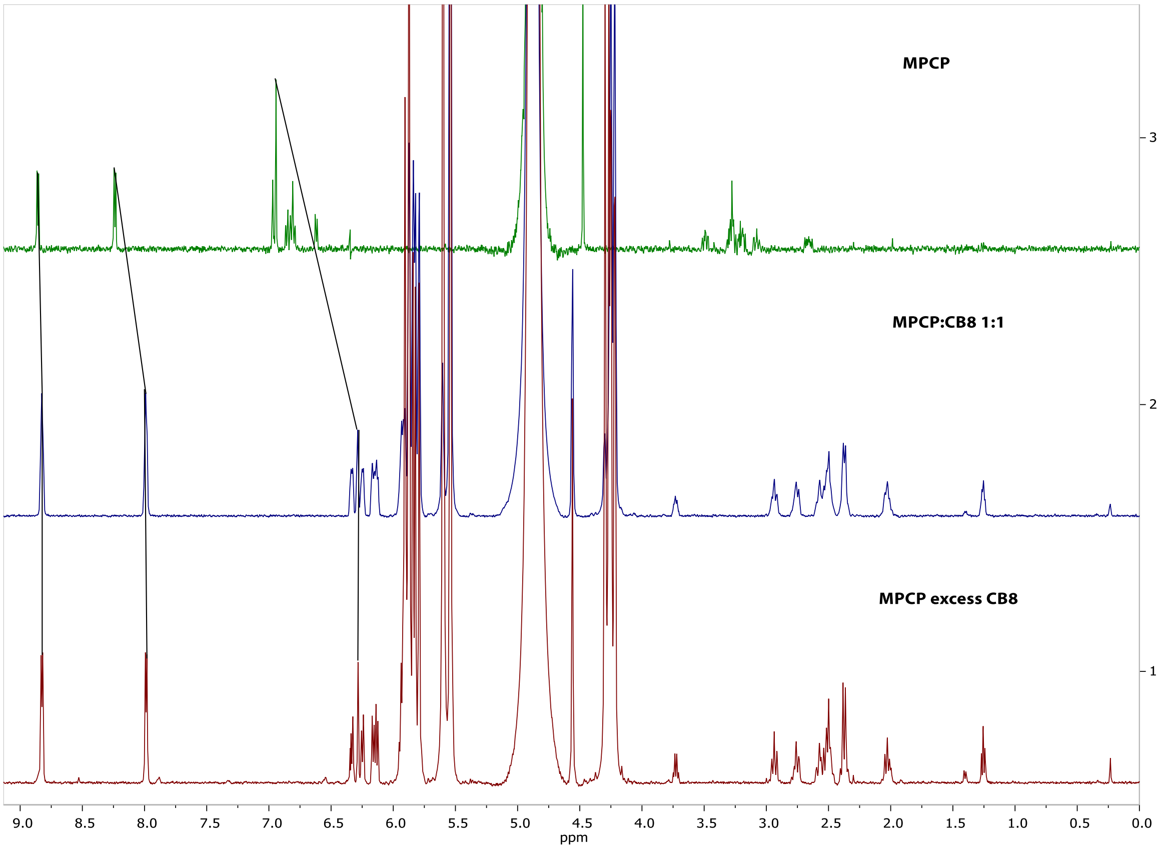

Fig. S 18: Stacked ^{1}H NMR (500 MHz, D_2O) spectra of pure **MPCP** (green), CB8:**MPCP** (1:1) (blue) and CB8:**MPCP** (excess CB8) (red).

3.3.4. Preparation of stock solutions for photophysical host-guest binding experiments

If not stated differently, Milli-Q water (type 1, Merck Millipore; >18.2 MΩ/cm at 25 °C) was used in all experiments. Phosphate buffered saline (PBS) was prepared from a tablet (GIPCO) together with Milli-Q water and kept in the fridge at +8°C for storage (pH=7.4). The stock solutions were prepared in Milli-Q water. The concentrations of the stock solutions of the chromophores BC (ε_{344} = 22,500 $M^{-1}cm^{-1}$)[5] and

MPCP (ε_{335} = 7,111 $M^{-1}cm^{-1}$, this work) were determined by UV-Vis absorption titration. The concentrations of the stock solutions of the hosts CB7 and CB8 were determined by UV-Vis absorption and fluorescence titration against Cobaltocenium and BC/**MPCP**, respectively. The concentration of the memantine stock solution was determined by a fluorescence-based indicator displacement assay using CB8⊃BC_2 as the chemosensor.

3.3.5. UV-Vis absorption spectroscopy

The electronic absorption spectra reveal, that the formation of an inclusion complex of **MPCP** with CB8 is accompanied by an isosbestic point at 366 nm (see **Fig. S 19**), which was determined by following Eq. S 7 to Eq. S 9.[6] The absorption maximum is bathochromically shifted upon inclusion complex formation, which is in line with the observed negative solvatochromism properties of **MPCP** (*vide supra*) and the nonpolar cavity of CB*n*.[7] Fluorescence titration experiments revealed a steep enhancement of the emission intensity upon addition of CB8 to an aqueous solution of **MPCP** comprising a hypsochromic shift.

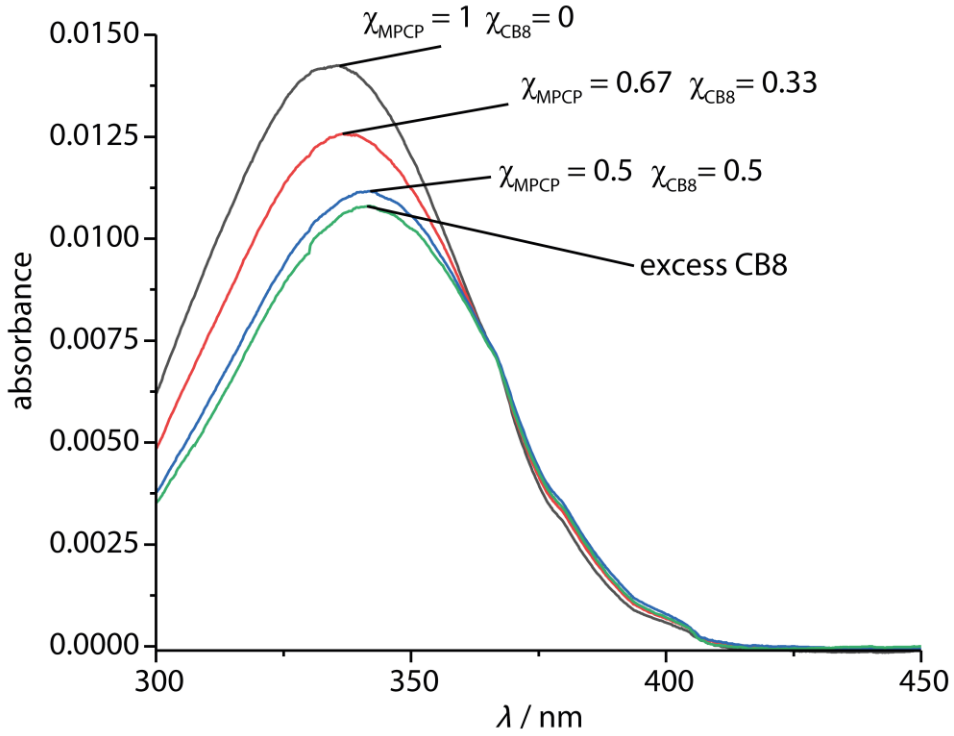

Fig. S 19: UV-Vis spectra of **MPCP** (c=18.1 µM) with increasing concentration of CB8. The molar fraction of CB8 is noted in the legend.

$$\bar{A}_k(\lambda) = k^{-1}\sum_{i=1}^{k} A_i(\lambda) \qquad \text{Eq. S 7}$$

$$\sigma_k(\lambda) = \left[k^{-1}\sum_{i=1}^{k}[A_i(\lambda) - \bar{A}_k(\lambda)]^2\right]^{\frac{1}{2}} \qquad \text{Eq. S 8}$$

$$\lambda_{isosbestic} = Minimum[\sigma_k(\lambda)] \qquad \text{Eq. S 9}$$

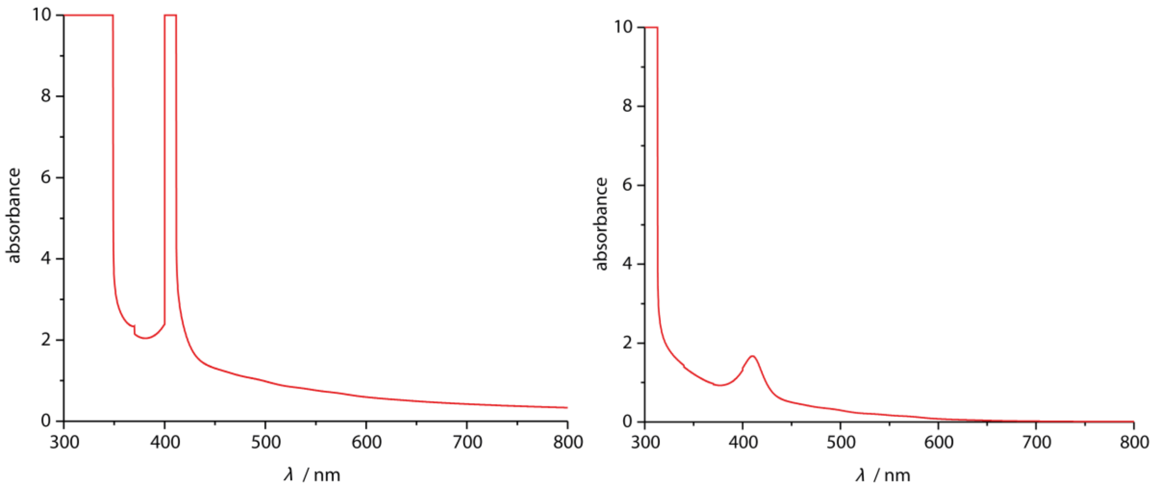

Fig. S 20: UV-Vis spectra of unfiltered bovine serum (left) and filtered bovine serum (0.22 mM sterile syringe filter).

3.3.6. Fluorescence spectroscopy

The binding isotherm resulting from a fluorescence titration of CB8 into a solution of **MPCP** indicates the very strong binding of the host and the guest. The intercept of the linear inclining curve at lower CB8 concentrations and the steady plateau at higher CB8 concentrations clearly display the formation of a 1:1 complex. Together with the depiction of the emission intensity as function of the molar fraction of CB8, the results of the host:guest fluorescence titrations are in agreement with the results of the mass spectrometry and NMR experiments. From the direct titration a binding constant > 10^9 was estimated, however, the binding of **MPCP** with CB8 is extremely strong, thus the binding isotherm of the direct host-guest titration is not useful for a meaningful fitting of the binding constant.

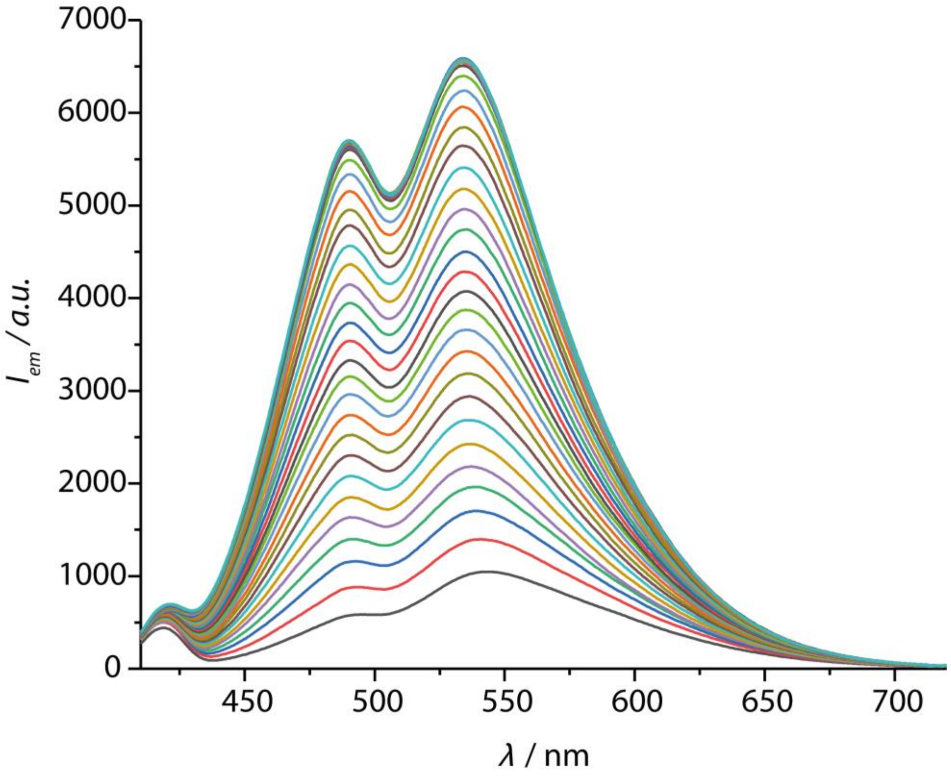

Fig. S 21: Corrected fluorescence spectra of **MPCP** (λ_{exc} = 366 nm, c = 1.9 µM) in water with increasing concentration of CB8 (0 – 3.1 µM).

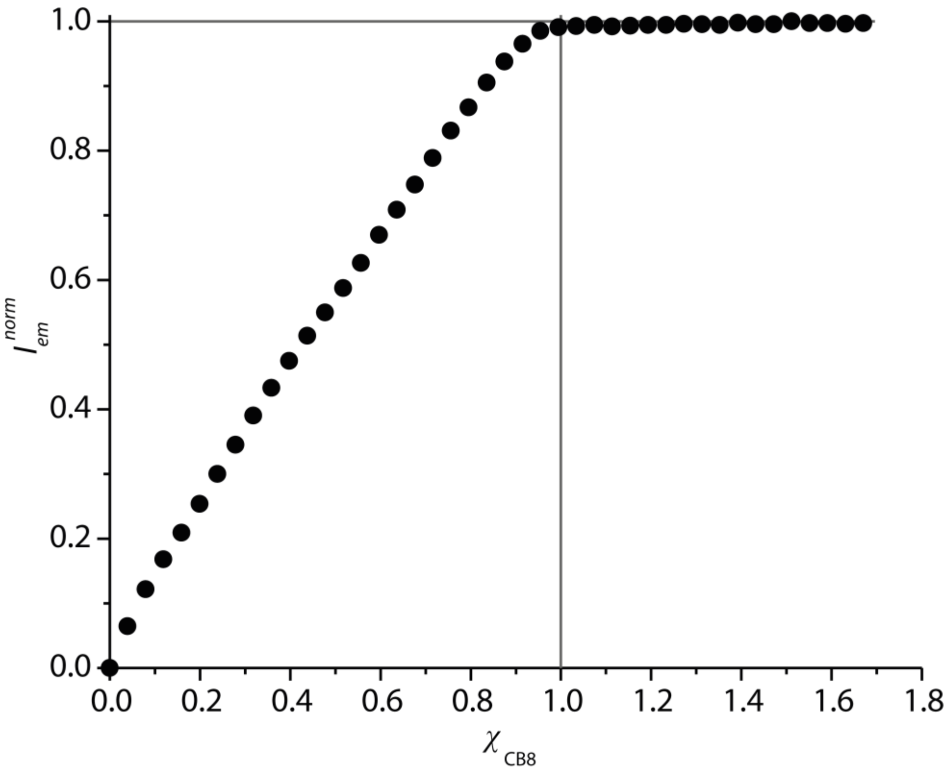

Fig. S 22: Normalized fluorescence intensity of **MPCP** (c = 1.9 µM) with varying CB8 molar fraction. The grey lines indicate the situation of an ideal 1:1 stoichiometry.

The bound-to-free signal ratio was determined from the titration curve to be I_{bound}:I_{free} = 6.8 by utilizing Eq. S 10.

$$\mathrm{I_{bound}}:\mathrm{I_{free}} = \frac{I_{\mathrm{CB[8]\supset MPCP}}}{I_{\mathrm{MPCP}}} = \frac{I_{max}^{tit}}{I_{min}^{tit}} \qquad \text{Eq. S 10}$$

Table S 2: Summary of the photophysical parameters of **MPCP** and its inclusion complex with CB8 under ambient conditions in water.

**Technique**	**Quantity / Unit**	**MPCP**	**CB8⊃MPCP**
**Absorption**	$\lambda_{abs,max}$ / nm	335	342
	$\varepsilon_{\lambda max}$ / $M^{-1}cm^{-1}$	7111 (± 190)	5653 (± 152)
	$\lambda_{isosbestic}$ / nm	366	
**Fluorescence**	$\lambda_{em,max}$ / nm	535	542
	$\lambda_{isoemissive}$ / nm	–	

3.3.7. Binding isotherms

In order to obtain binding isotherms suitable for a meaningful fitting of the binding constant, competitive fluorescence titration experiments were carried out. Firstly, the binding constants of the complex formation of CB8⊃BC_2 were determined by fluorescence titration experiments with a subsequent binding model on the basis of Eqs. S 12, S 13 (right), S 14 (right) and are in very good agreement with the literature values from Biczok *et al.* (see **Table S 3**).[8]
Secondly, the binding isotherms of ultra-high affinity binders such as **MPCP** and **Mem** were obtained by an IDA with CB8⊃BC_2. The isotherms were fitted to a binding model derived from the Eqs. S 12 - 15 (numerical solutions by Mathematica) and the corresponding K_a values were determined as the mean (± standard deviation) of 10 independent titration experiments (see ESI for titration data).

$$H + G \leftrightarrows HG \qquad H + D \leftrightarrows HD \qquad HD + D \leftrightarrows HDD$$ Eq. S 11

$$I_{signal}^{em} = \alpha_{HD}[HD] + \alpha_{HDD}[HDD] + \alpha_D[D]$$ Eq. S 12

$$K_a^{HG} = \frac{[\mathrm{H}G]}{[\mathrm{H}]\cdot[G]} \quad K_a^{HD} = \frac{[\mathrm{H}D]}{[\mathrm{H}]\cdot[D]} \quad K_a^{HDD} = \frac{[\mathrm{H}DD]}{[\mathrm{HD}]\cdot[D]}$$ Eq. S 13

$$[G]_0 = [G] + [HG] \quad [D]_0 = [D] + [HD] + 2[HDD]$$ Eq. S 14

$$[H]_0 = [H] + [HD] + [HDD] + [HG]$$ Eq. S 15

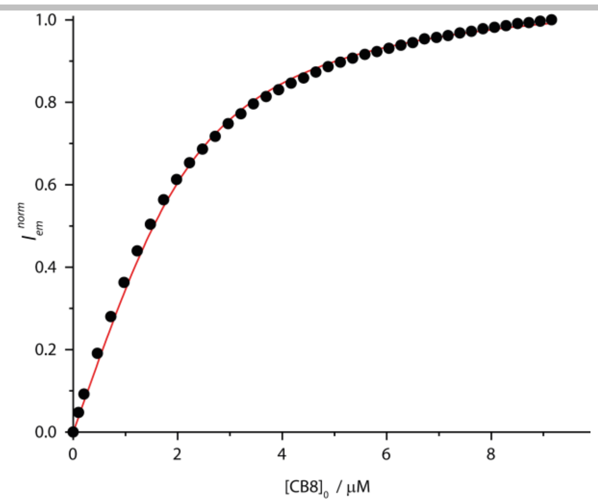

Fig. S 23: Titration experiments of BC (c = 2 µM) with increasing concentration of CB8 in PBS at T = 25°C. The black dots display the obtained data point from the titration and the red line represents the fitted data. λ_{exc} = 462 nm, λ_{em} = 548 nm

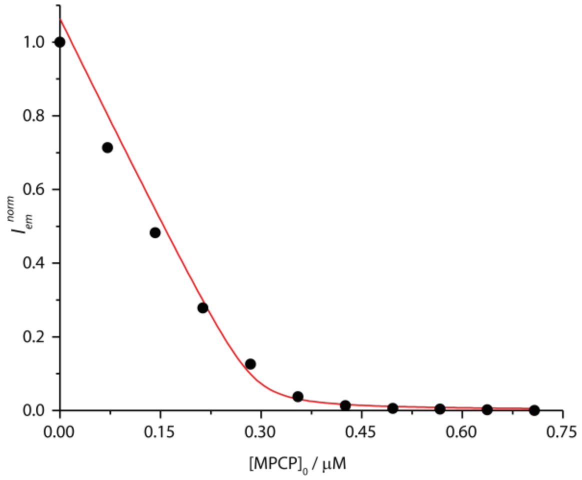

Fig. S 24: Indicator-displacement assays of CB8⊃BC$_2$ (CB8: c = 0.57 µM; BC: c = 59.2 µM) with increasing concentration of **MPCP** in Milli-Q water at T = 25°C (see main text for PBS). The black dots display the obtained data point from the titration and the red line represents the fitted data. λ_{exc} = 462 nm, λ_{em} = 548 nm

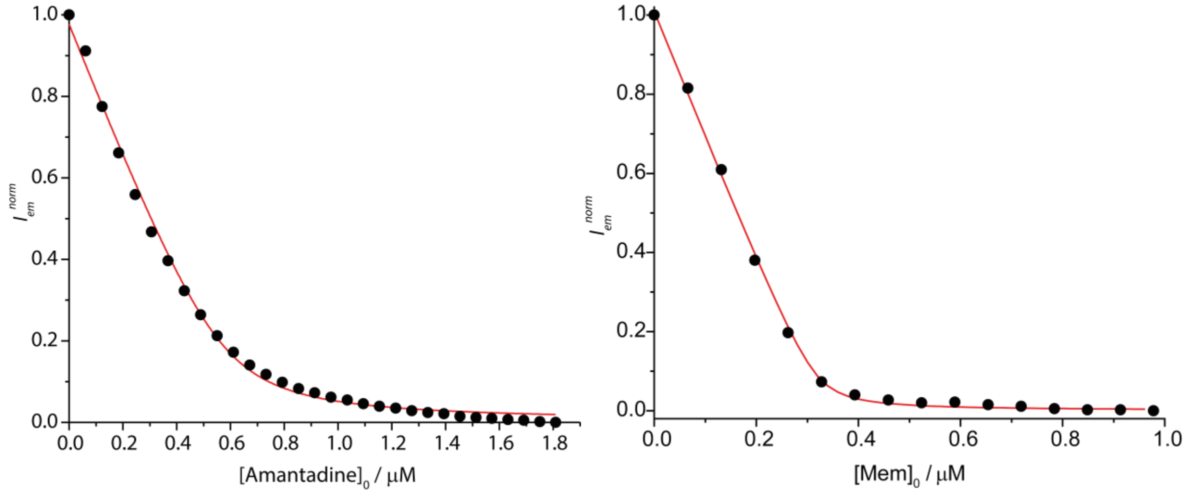

Fig. S 25: Indicator-displacement assays of CB8⊃BC$_2$ (left: CB8: c = 0.59 µM; BC: c = 3.6 µM; Amantadine, right: CB8: c = 0.32 µM; BC: c = 33.7 µM; Memantine) with increasing concentration of analyte in PBS at T = 25°C. The black dots display the obtained data point from the titration and the red line represents the fitted data. λ_{exc} = 462 nm, λ_{em} = 548 nm

Table S 3: Summary of the binding constants determined by fluorescence titration experiments

Complex	Medium	K_a	log K_a
CB8⊃BC₂	water - lit[8]	2.09 (± 0.65) · 10^{13} M^{-2}	13.32 (± 0.13)
	water	1.02 (± 0.33) · 10^{13} M^{-2}	13.01 (± 0.14)
	PBS	2.41 (± 1.22) · 10^{11} M^{-2}	11.38 (± 0.22)
	NaAc pH = 4.75	2.32 (± 0.78) · 10^{13} M^{-2}	13.37 (± 0.15)
CB8⊃Mem	water	8.28 (± 0.38) · 10^{12} M^{-1}	12.92 (± 0.02)
	PBS	1.33 (± 0.47) · 10^{11} M^{-1}	11.12 (± 0.15)
	NaAc pH = 4.75	1.70 (± 0.53) · 10^{13} M^{-1}	13.23 (± 0.13)
CB8⊃MPCP	water	3.89 (± 0.99) · 10^{12} M^{-1}	12.59 (± 0.02)
	PBS	7.55 (± 1.68) · 10^{10} M^{-1}	10.88 (± 0.10)
CB8⊃Amantadine	water	5.55 (± 0.75) · 10^{9} M^{-1}	9.74 (± 0.06)
	PBS	3.55 (± 0.45) · 10^{8} M^{-1}	8.55 (± 0.06)

T = 25°C, the depicted binding constants (K_a) are the average of 10 independent binding experiments and the standard deviation is used as error

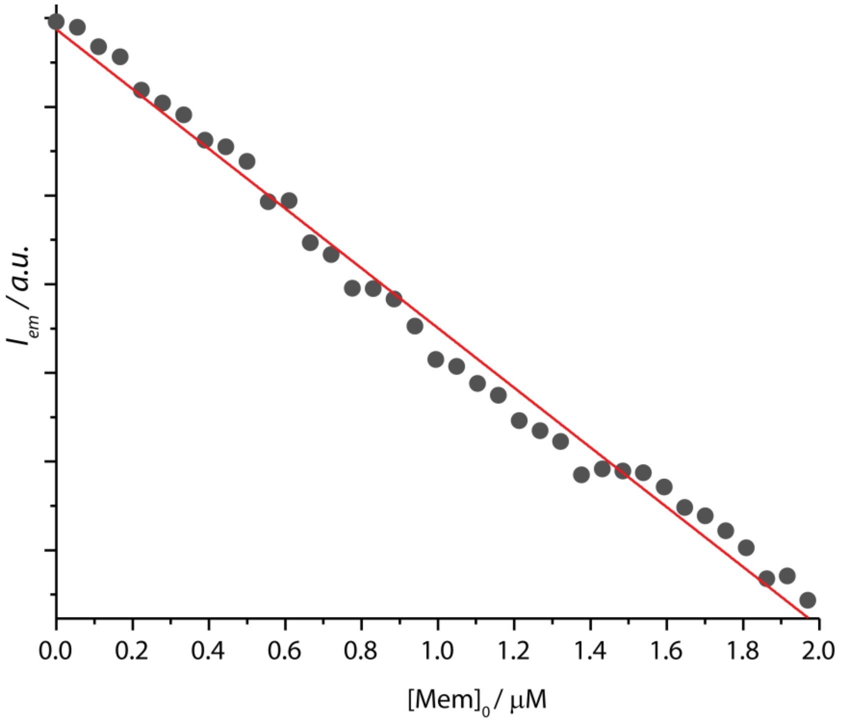

Fig. S 26: Fluorescence based IDA of CB8⊃**MPCP** (λ_{exc} = 335 nm, λ_{em} = 535 nm, c = 9.67 µM) with increasing concentration of **Mem** (0 – 2 µM) in PBS at T = 25°C. Slope = -133 a.u. μM^{-1}

3.3.8. Indicator displacement assay in blood serum

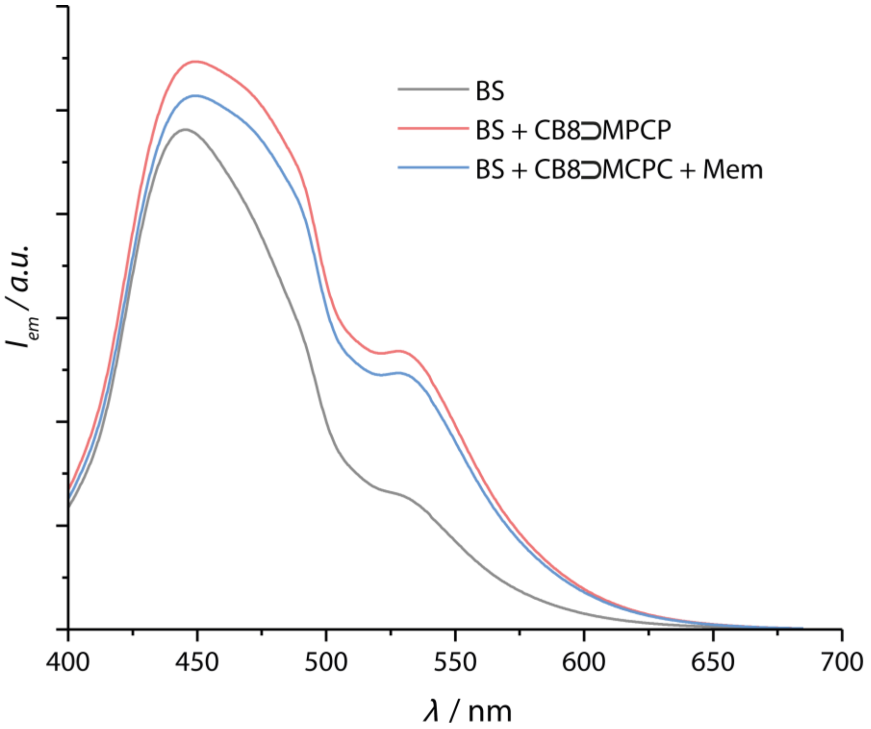

Fig. S 27: Fluorescence based IDA of CB8⊃**MPCP** (conc. = 9.9 µM, λ_{exc} = 335 nm, λ_{em} = 535 nm) with **Mem** (10 µM)in bovine blood serum at T = 25°C.

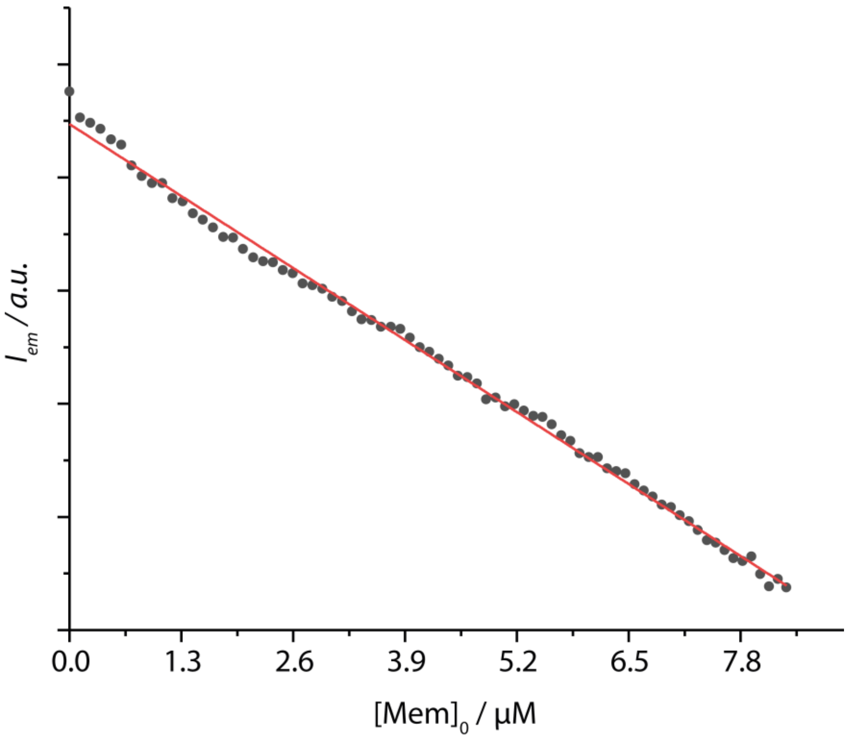

Fig. S 28: Fluorescence based IDA of CB8⊃**MPCP** (λ_{exc} = 335 nm, λ_{em} = 535 nm, c = 9.9 µM) with increasing concentration of **Mem** (0 – 8.3 µM) in bovine blood serum at T = 25°C.

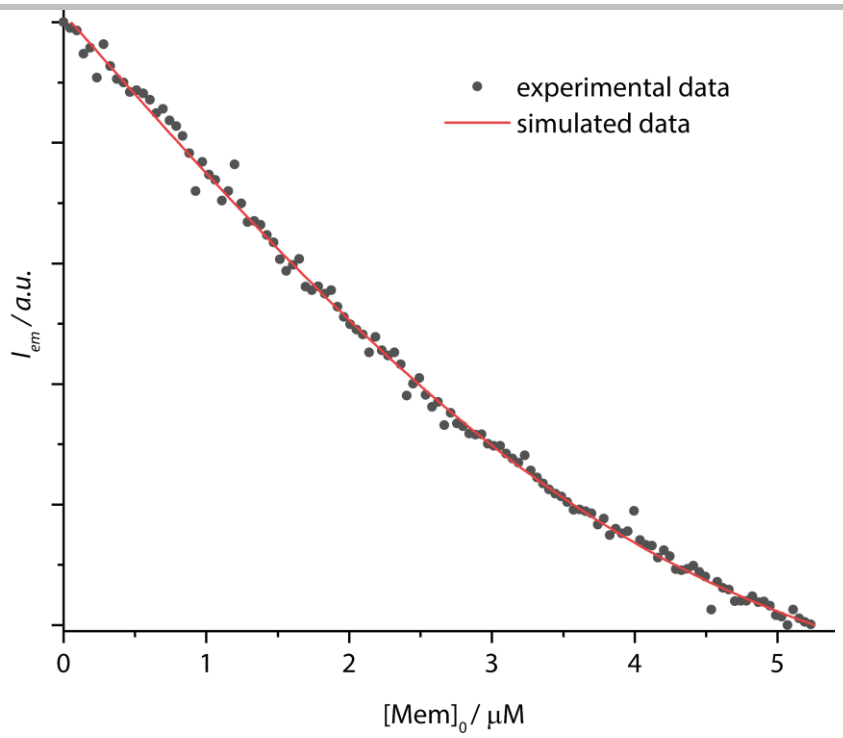

Fig. S 29: Fluorescence based IDA of CB8⊃**MPCP** (λ_{exc} = 335 nm, λ_{em} = 535 nm, c = 5.13 μM) with increasing concentration of **Mem** (0 – 5.3 μM) in human blood serum at T = 25°C (black dots) alongside the simulation for this experiment.

Table S 4: Result of three independent IDA calibration measurements in bovine serum (BS) samples with CB8⊃**MPCP** vs. **Mem**

Sample	*Slope / μM^{-1}*
BS-1	-132.2
BS-2	-110.8
BS-3	-125.6
Mean	-122.9
Error	10.95

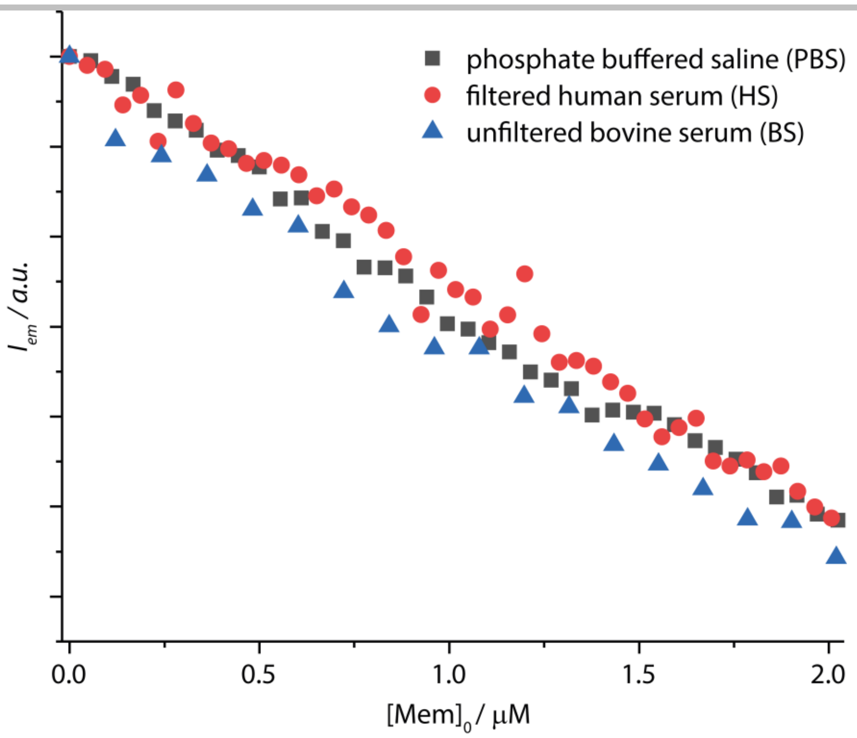

Fig. S 30: Fluorescence based IDA of CB8⊃**MPCP** (λ_{exc} = 335 nm, λ_{em} = 535 nm, c = 5.13 µM) with increasing concentration of **Mem** (0 – 2 µM) in human and bovine blood sera and PBS (c = 9.67 µM)at T = 25°C. The offset of the ordinate was adjusted compensating the different medium dependent background emission and inner filter effects.

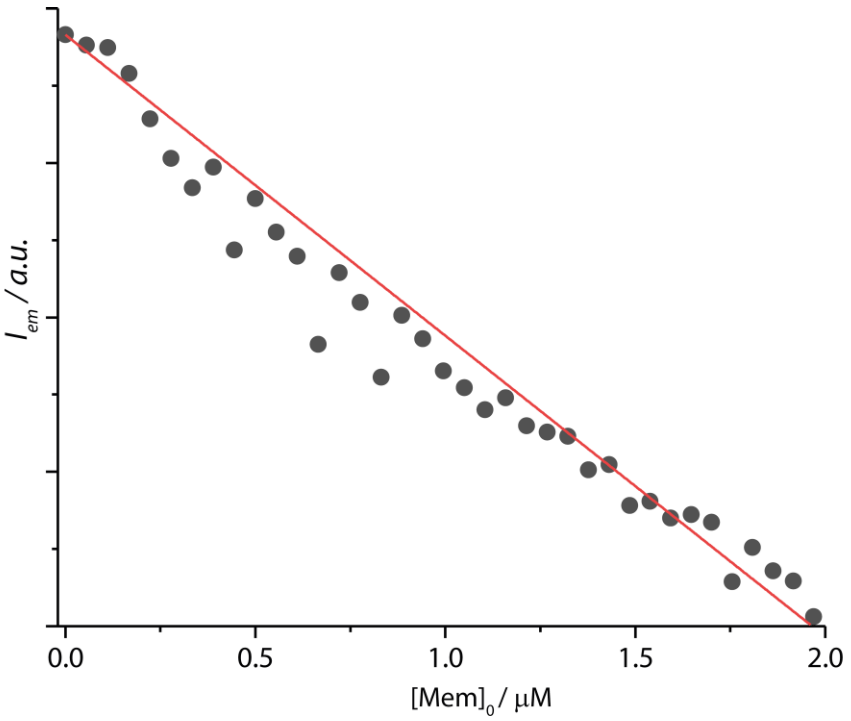

Fig. S 31: Fluorescence based IDA of CB8⊃**MPCP** (λ_{exc} = 335 nm, λ_{em} = 535 nm, c = 9.9 µM) with increasing concentration of **Mem** (0 – 2 µM) in pig blood serum at T = 25°C.

3.4. Host-guest inclusion complex formation of MPCP with CB7

3.4.1. DFT calculations

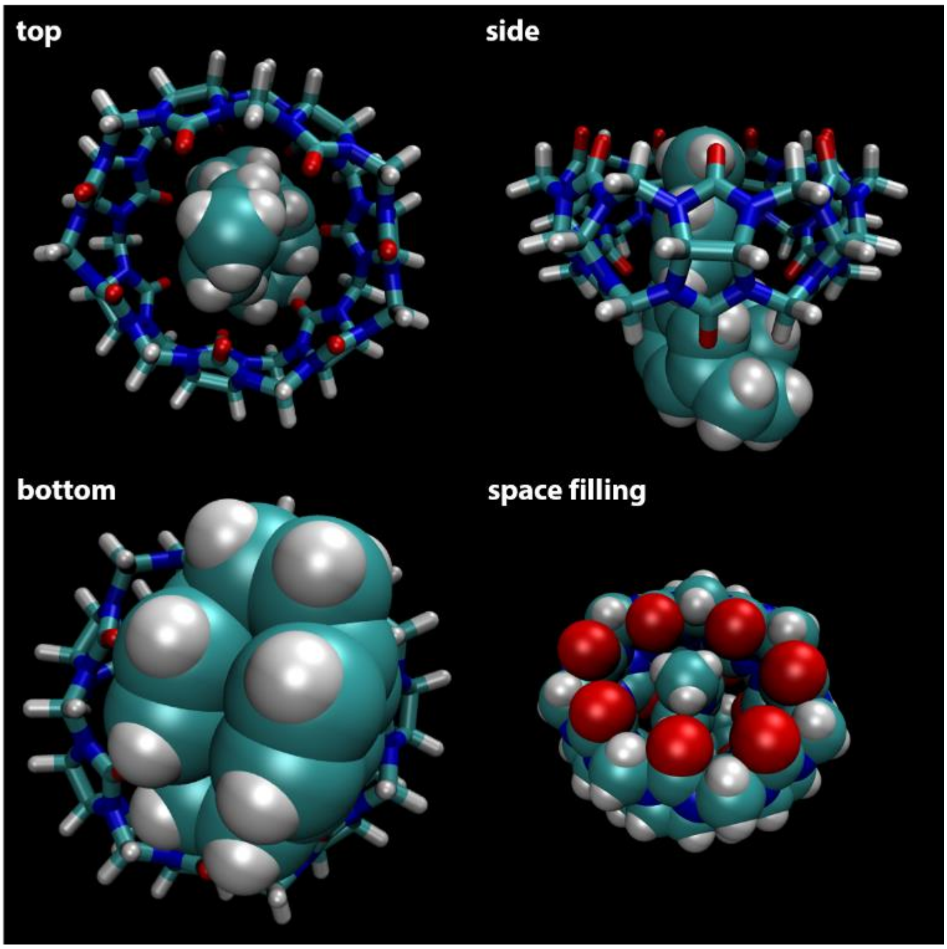

Fig. S 32: DFT ground-state optimized host:guest complex structure of CB7⊃**MPCP** in top, side and bottom view featuring CB7 in licorice and **MPCP** in space filling representation. On the bottom right both entities are displayed in space filling style from a top perspective. DFT calculations were carried out utilizing the hybrid functional B3LYP with the standard valence basis set 6-31G(d,p) for C,H,N and O. It is evident that only the smaller pyridinium moiety and not the PCP is engulfed by the CB7 cavity, in agreement with the experimental results from the ^{1}H NMR measurements.

3.4.2. NMR spectroscopy

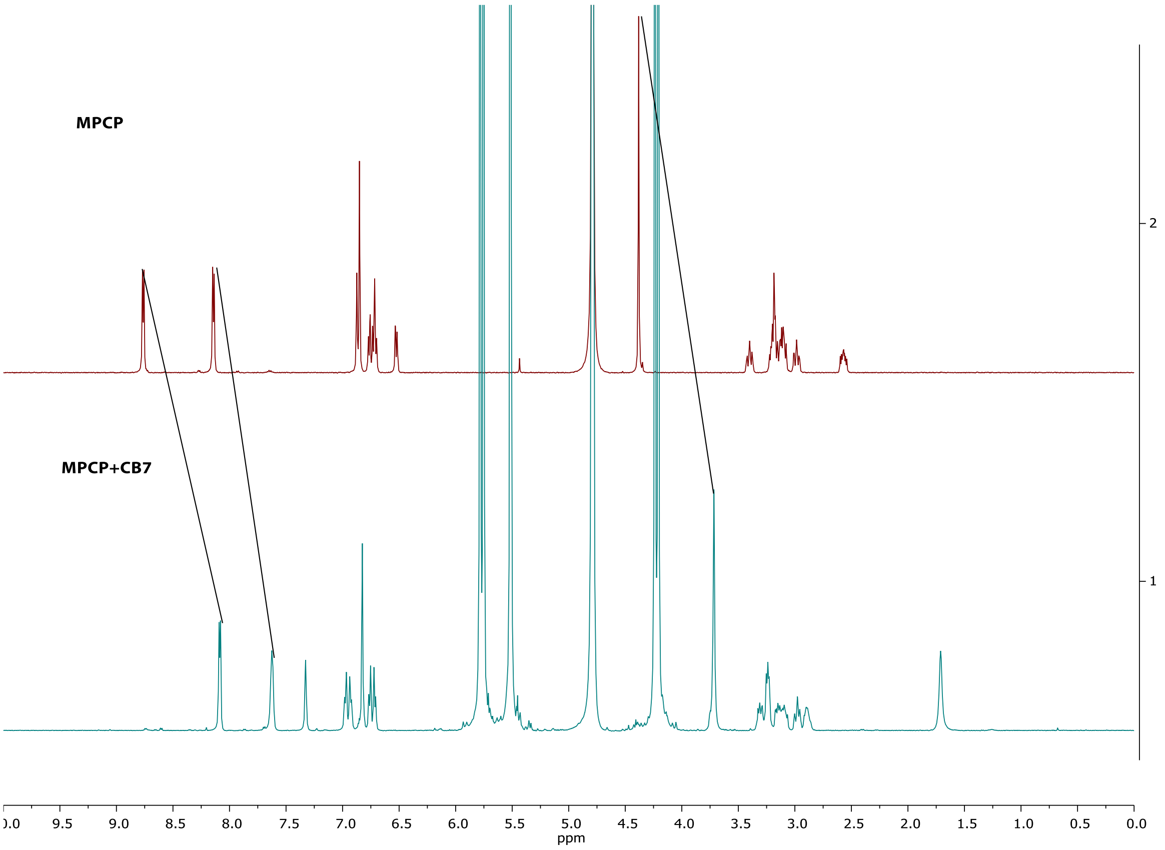

Fig. S 33: Stacked ^{1}H NMR (500 MHz, D_2O) spectra of pure **MPCP** (red and CB7:**MPCP** (excess CB7) (cyan). The NMR data suggests that only the smaller pyridinium moiety and not the PCP is engulfed by the CB7 cavity, in agreement with the modeling results.

3.5. ^{1}H and ^{13}C NMR spectra of the MPCP precursors and of MPCP

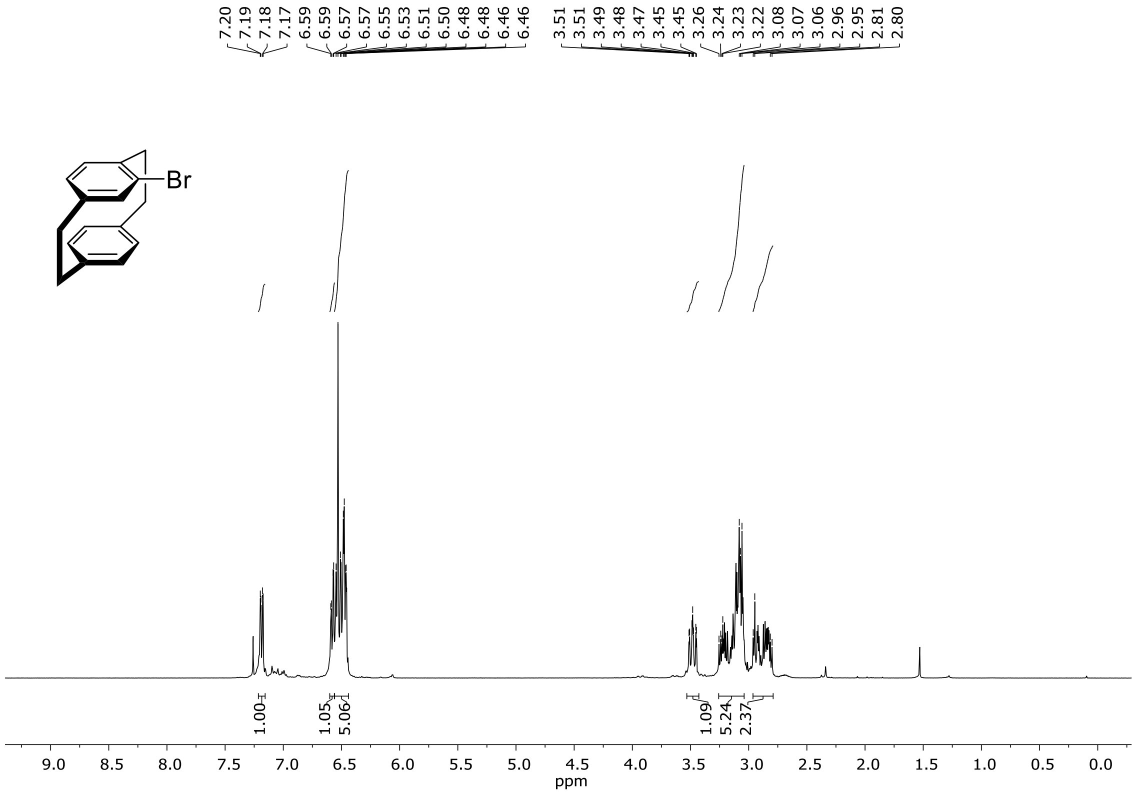

Fig. S 34: ^{1}H NMR (400 MHz, $CDCl_3$) spectrum of (*rac*)-4-bromo[2.2]paracyclophane (**BrPCP**).

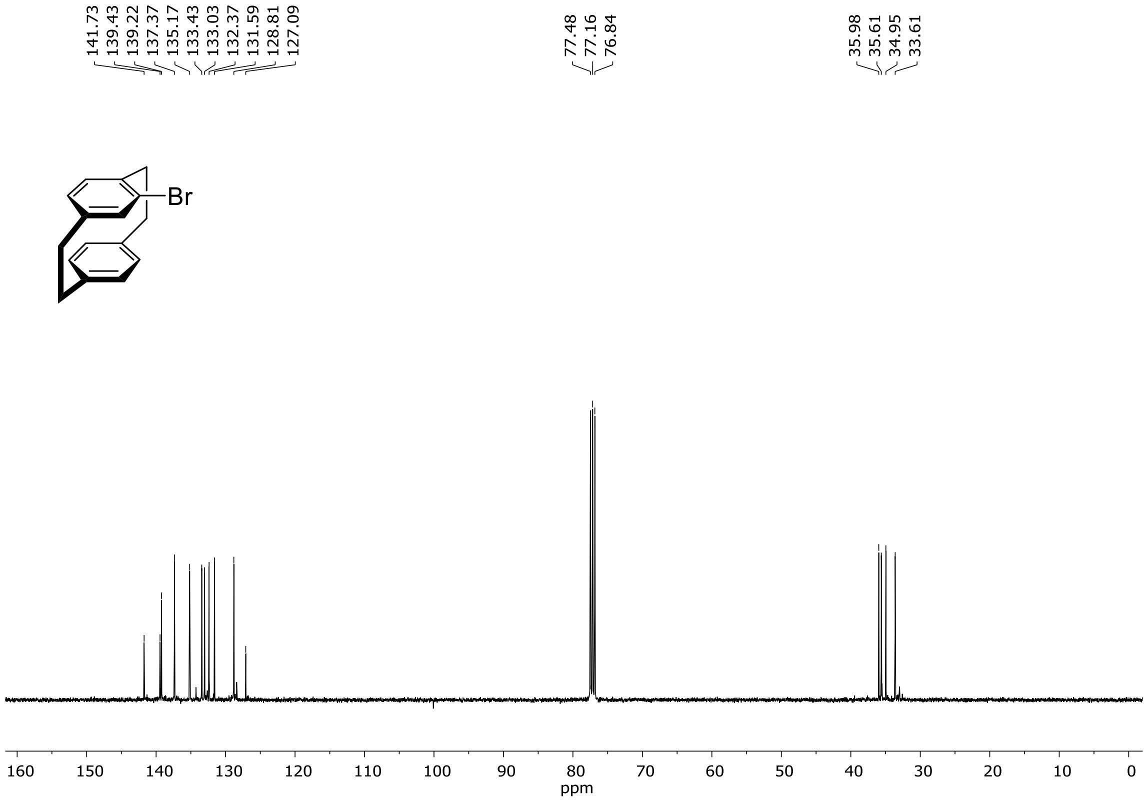

Fig. S 35: ^{13}C NMR (100 MHz, $CDCl_3$) spectrum of (*rac*)-4-bromo[2.2]paracyclophane (**BrPCP**).

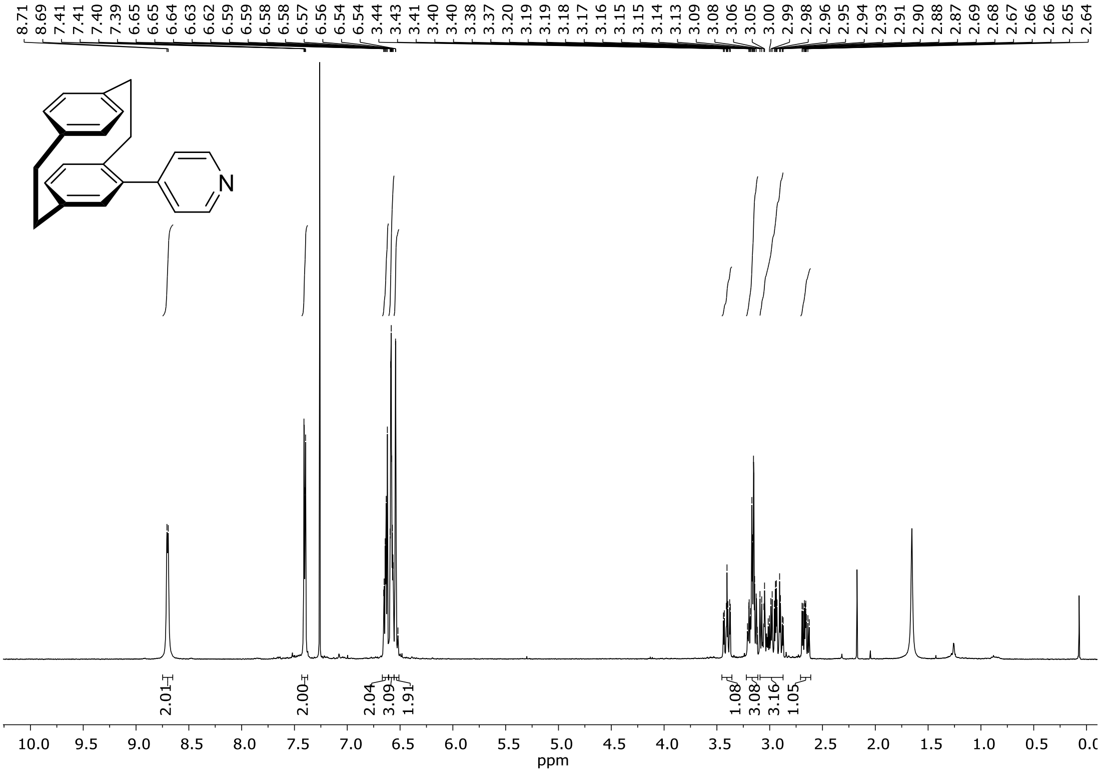

Fig. S 36: ^{1}H NMR (400 MHz, $CDCl_3$) spectrum of *(rac)*-4-pyridyl[2.2]paracyclophane (**PyPCP**).

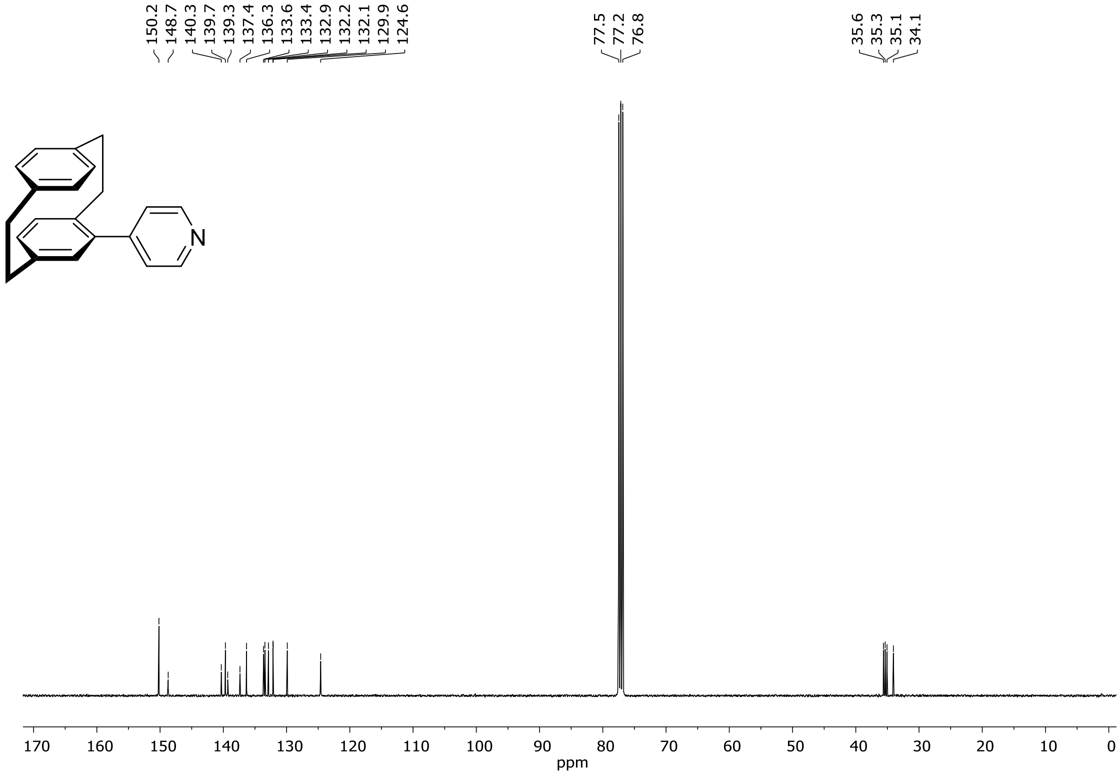

Fig. S 37: ^{13}C NMR (100 MHz, $CDCl_3$) spectrum of (*rac*)-4-pyridyl[2.2]paracyclophane (**PyPCP**).

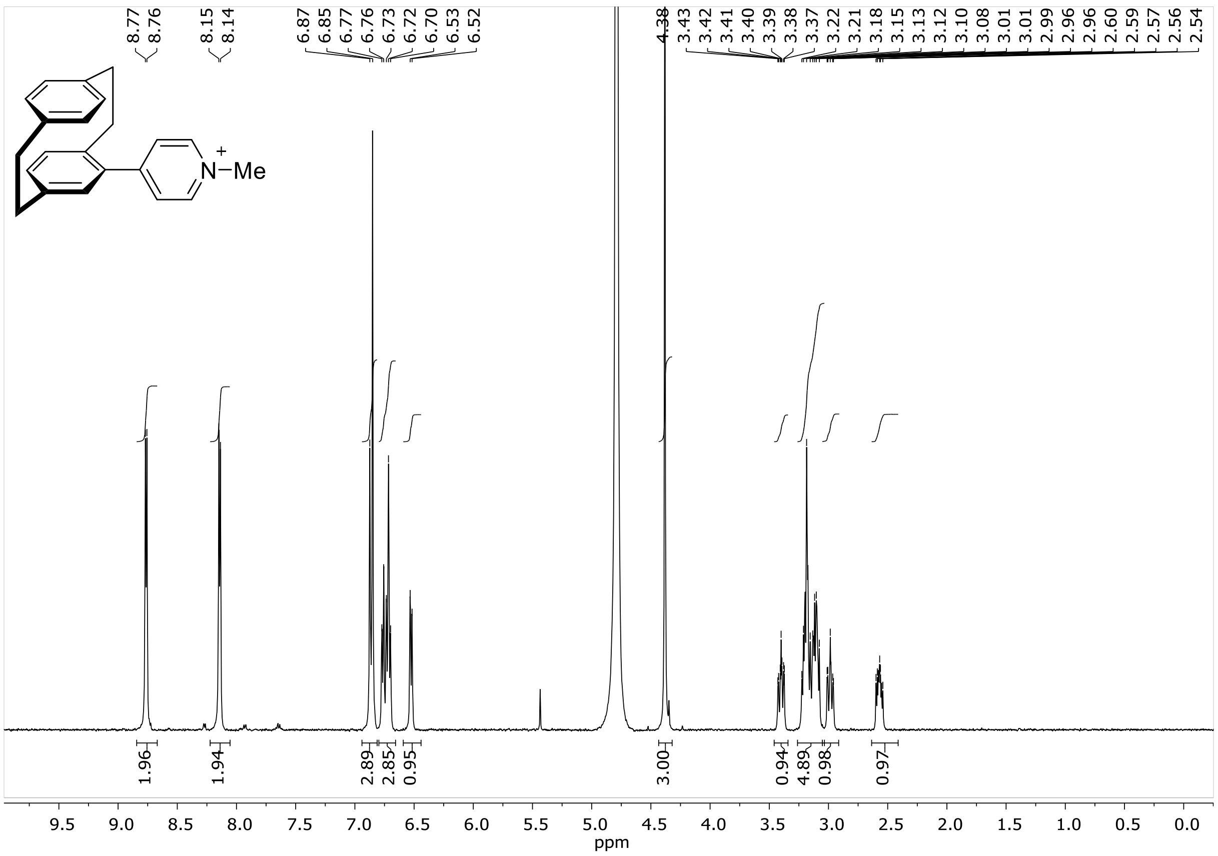

Fig. S 38: ^{1}H NMR (500 MHz, D_2O) spectrum of (*rac*)-*N*-methyl-4-pyridinylium[2.2]paracyclophane (**MPCP**).

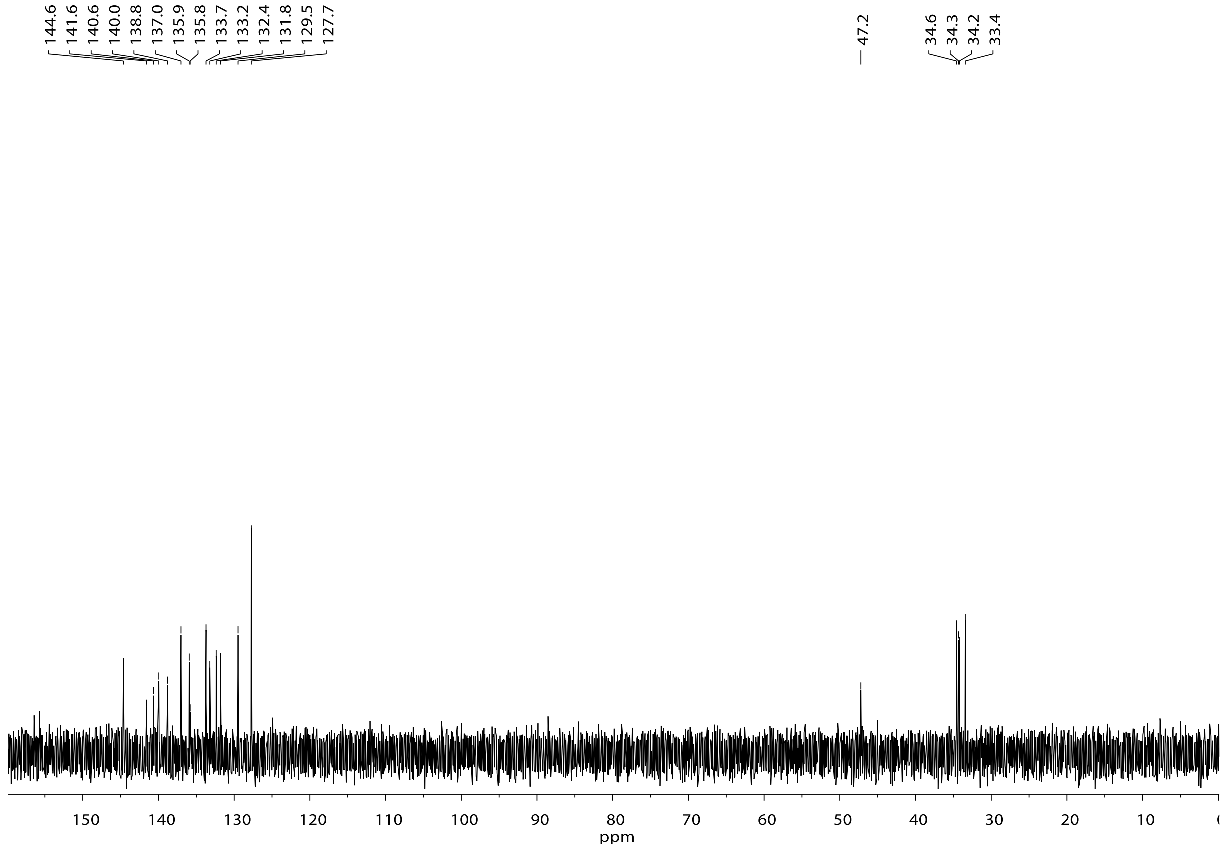

Fig. S 39: ^{13}C NMR (126 MHz, D_2O) spectrum of (*rac*)-*N*-methyl-4-pyridinylium[2.2]paracyclophane (**MPCP**).

4. References

1. L. Ernst, *Liebigs Ann.*, 1995, DOI: 10.1002/jlac.199519950105, 13—17.
2. C. Braun, E. Spuling, N. B. Heine, M. Cakici, M. Nieger and S. Bräse, *Adv. Synth. Catal.*, 2016, **358**, 1664-1670.
3. C. Reichardt, *Chem. Rev.*, 1994, **94**, 2319-2358.
4. M. J. Frisch, G. W. Trucks, H. B. Schlegel, G. E. Scuseria, M. A. Robb, J. R. Cheeseman, G. Scalmani, V. Barone, G. A. Petersson, H. Nakatsuji, X. Li, M. Caricato, A. V. Marenich, J. Bloino, B. G. Janesko, R. Gomperts, B. Mennucci, H. P. Hratchian, J. V. Ortiz, A. F. Izmaylov, J. L. Sonnenberg, Williams, F. Ding, F. Lipparini, F. Egidi, J. Goings, B. Peng, A. Petrone, T. Henderson, D. Ranasinghe, V. G. Zakrzewski, J. Gao, N. Rega, G. Zheng, W. Liang, M. Hada, M. Ehara, K. Toyota, R. Fukuda, J. Hasegawa, M. Ishida, T. Nakajima, Y. Honda, O. Kitao, H. Nakai, T. Vreven, K. Throssell, J. A. Montgomery Jr., J. E. Peralta, F. Ogliaro, M. J. Bearpark, J. J. Heyd, E. N. Brothers, K. N. Kudin, V. N. Staroverov, T. A. Keith, R. Kobayashi, J. Normand, K. Raghavachari, A. P. Rendell, J. C. Burant, S. S. Iyengar, J. Tomasi, M. Cossi, J. M. Millam, M. Klene, C. Adamo, R. Cammi, J. W. Ochterski, R. L. Martin, K. Morokuma, O. Farkas, J. B. Foresman and D. J. Fox, *Gaussian, Inc*., Wallingford CT, 2016.
5. K. Bhadra and G. S. Kumar, *Biochim. Biophys. Acta.*, 2011, **1810**, 485-496.
6. P. O. Vardevanyan, V. L. Élbakyan, M. A. Shahinyan, M. V. Minasyants, M. A. Parsadanyan and N. S. Sahakyan, *J. Appl. Spectrosc.*, 2015, **81**, 1060-1063.
7. K. I. Assaf and W. M. Nau, *Chem. Soc. Rev.*, 2015, **44**, 394-418.
8. Z. Miskolczy and L. Biczok, *Phys. Chem. Chem. Phys.*, 2014, **16**, 20147-20156.
