# Supplementary material for: Rational design and implementation of a cucurbit[8]uril-based indicator-displacement assay for application in blood serum
Source: Chem Sci. 2019 Jun 4;10(27):6584–93. doi: 10.1039/c9sc00705a (PMC6628674; doi:10.1039/c9sc00705a)
Supplement: Supplementary file 2 [file SC-010-C9SC00705A-s002.pdf]

Electronic Supplementary Material (ESI) for Chemical Science.
This journal is © The Royal Society of Chemistry 2019

A bloody affair: Novel indicator dye and Cucurbit[8]uril (CB8) conjugate for detection of supramolecules in blood

Low-cost, robust artificial receptors like CB8 can be used as "reporters" for detecting and monitoring drug levels.

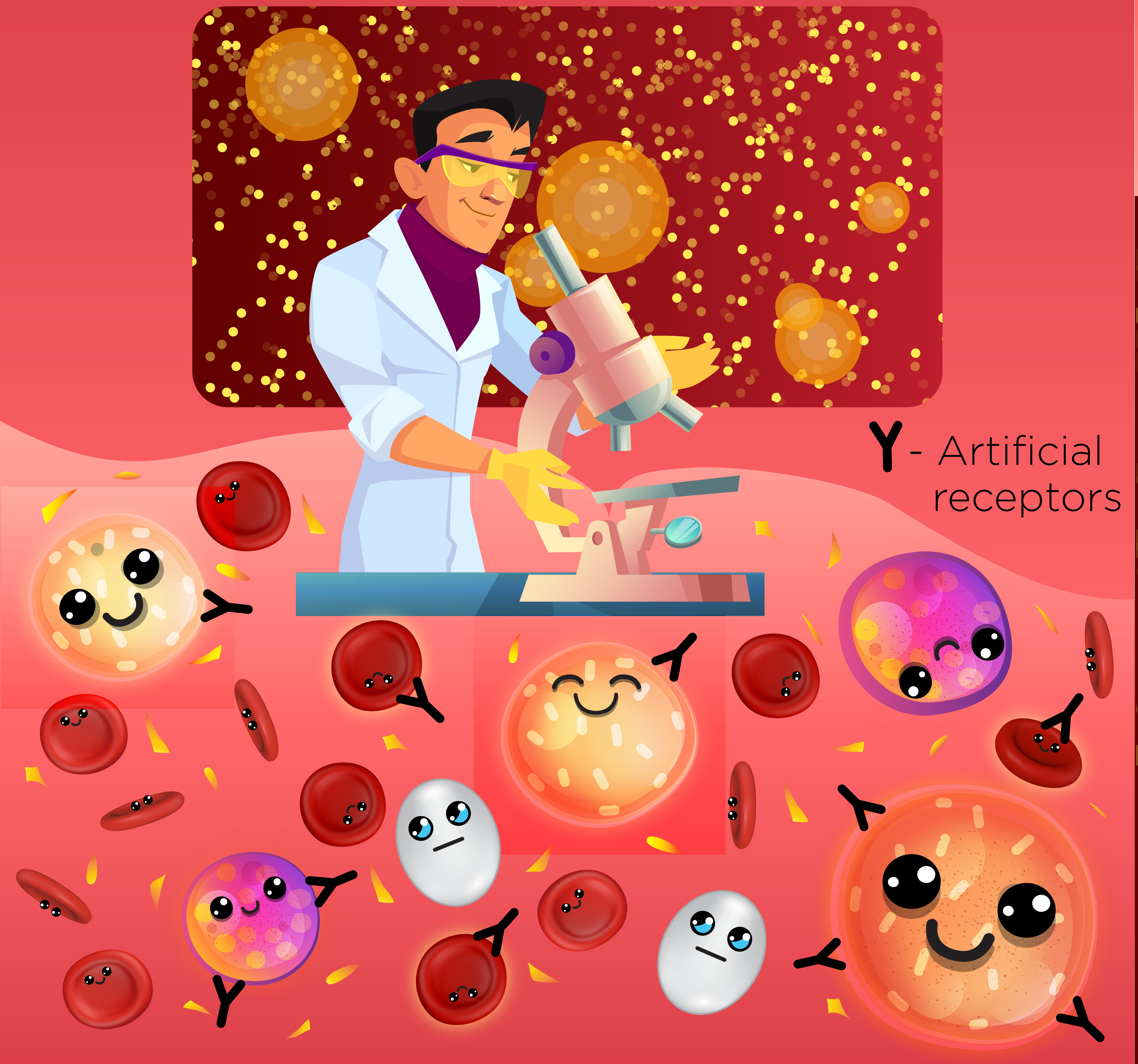

However, numerous molecules are present in blood, which compete to bind with these reporters reducing the effectiveness of such sensing methods.

Synthesis of novel indicator dye in conjunction with receptor CB8

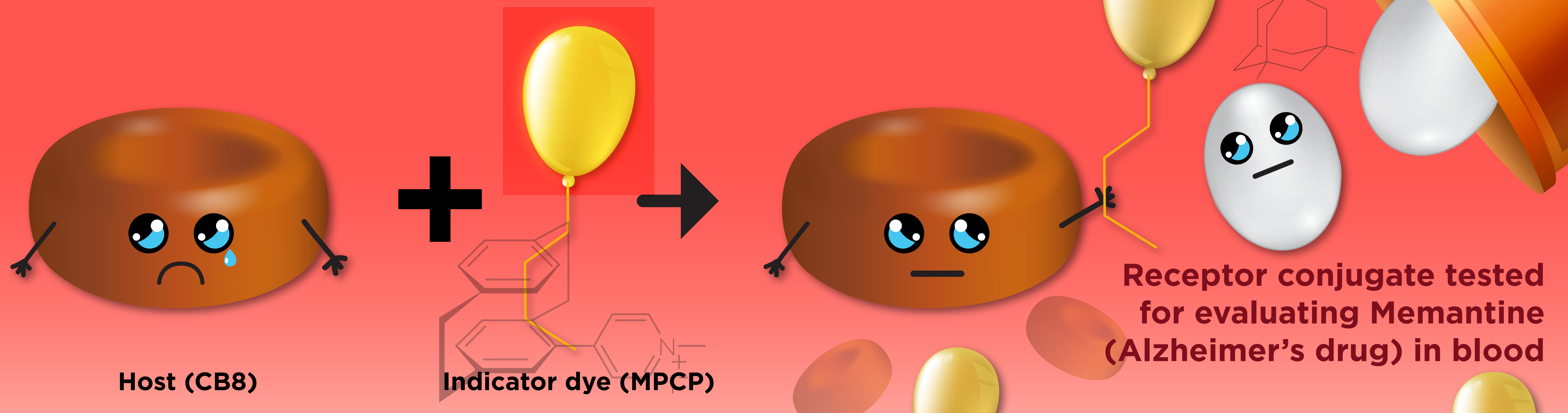

Receptor conjugate tested for evaluating Memantine (Alzheimer's drug) in blood

Benefits of this detection:

- High binding strength
- Accurate estimation
- Purely synthetic hosts
- Sensitive to sample variations
- Fast and inexpensive

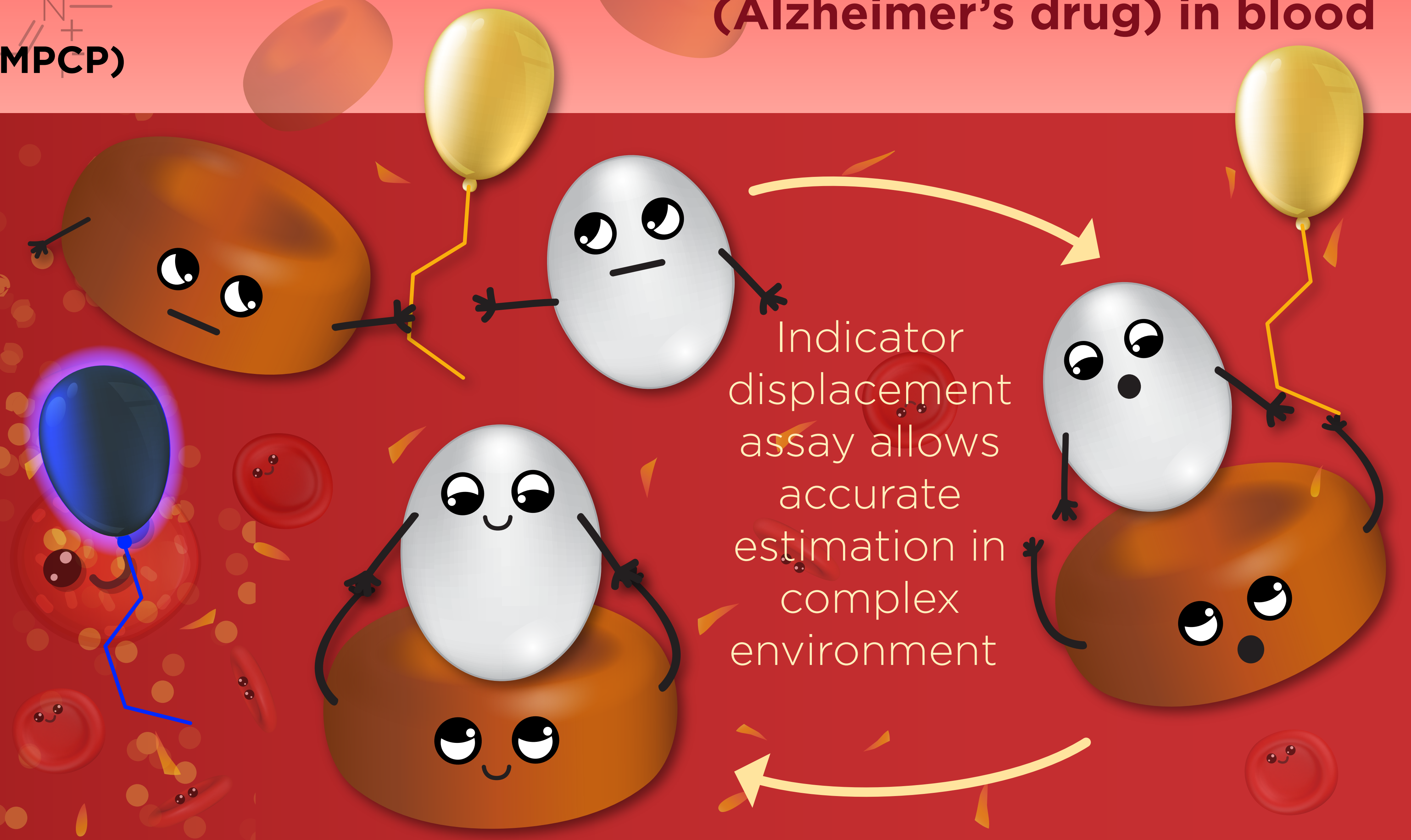

CB8-based chemosensing ensemble exhibits excellent sensing capabilities and could be used for developing diverse blood serum-based supramolecular assays.

Chemical Science

PICK OF THE WEEK

Rational Design and Implementation of a Cucurbit[8]uril based Indicator-Displacement Assay for Application in Blood Serum
Sinn *et al.* (2019)
DOI: 10.1039/C9SC00705A

ROYAL SOCIETY OF CHEMISTRY
